# Granulosa cell-derived extracellular vesicles mitigate the detrimental impact of thermal stress on bovine oocytes and embryos

**DOI:** 10.3389/fcell.2023.1142629

**Published:** 2023-04-06

**Authors:** Nico G. Menjivar, Ahmed Gad, Samuel Gebremedhn, Soham Ghosh, Dawit Tesfaye

**Affiliations:** ^1^ Animal Reproduction and Biotechnology Laboratory (ARBL), Department of Biomedical Sciences, College of Veterinary Medicine and Biomedical Sciences, Colorado State University, Fort Collins, CO, United States; ^2^ Department of Animal Production, Faculty of Agriculture, Cairo University, Giza, Egypt; ^3^ Genus Plc, Deforest, WI, United States; ^4^ Cellular Engineering and Mechanobiology Laboratory (CEML), Department of Mechanical Engineering, Translational Medicine Institute (TMI), Colorado State University, Fort Collins, CO, United States

**Keywords:** extracellular vesicles, granulosa cells, cumulus-oocyte complex, oocyte maturation, heat stress, embryo development

## Abstract

Climate change-induced global warming results in rises in body temperatures above normal physiological levels (hyperthermia) with negative impacts on reproductive function in dairy and beef animals. Extracellular vesicles (EVs), commonly described as nano-sized, lipid-enclosed complexes, harnessed with a plethora of bioactive cargoes (RNAs, proteins, and lipids), are crucial to regulating processes like folliculogenesis and the initiation of different signaling pathways. The beneficial role of follicular fluid-derived EVs in inducing thermotolerance to oocytes during *in vitro* maturation (IVM) has been evidenced. Here we aimed to determine the capacity of *in vitro* cultured granulosa cell-derived EVs (GC-EVs) to modulate bovine oocytes’ thermotolerance to heat stress (HS) during IVM. Moreover, this study tested the hypothesis that EVs released from thermally stressed GCs (S-EVs) shuttle protective messages to provide protection against subsequent HS in bovine oocytes. For this, sub-populations of GC-EVs were generated from GCs subjected to 38.5°C (N-EVs) or 42°C (S-EVs) and supplemented to cumulus-oocyte complexes (COCs) matured *in vitro* at the normal physiological body temperature of the cow (38.5°C) or HS (41°C) conditions. Results indicate that S-EVs improve the survival of oocytes by reducing ROS accumulation, improving mitochondrial function, and suppressing the expression of stress-associated genes thereby reducing the severity of HS on oocytes. Moreover, our findings indicate a carryover impact from the addition of GC-EVs during oocyte maturation in the development to the blastocyst stage with enhanced viability.

## 1 Introduction

One of the leading, most consequential interferences to a homeostatic reproductive environment in dairy and beef cattle is elevated summer HS, a prime determinant of fertility and reproductive function (Boni, 2019; Wolfenson and Roth, 2019). Hyperthermia, the abnormal thermal state induced through failed regulatory mechanisms of the body, occurs when endogenous heat production supersedes the subsequent heat loss to the environment (Hansen, 2009). Thermal stress is known to compromise follicular development, ovarian functionality, fertilization, and subsequent early embryonic development (Badinga et al., 1993; Wolfenson et al., 1995; Rivera and Hansen, 2001; Sartori et al., 2002). Of specific interest in reduced fecundity is HS-induced damage to proper oocyte function. It is important to note that oocytes act spontaneously and independently of one another. However, elevated thermal environments are in part responsible for deleterious ramifications on the molecular, functional, and cellular changes to oocytes and their surrounding cumulus cells (CCs) (Abdelnour et al., 2020).

Mammalian oocytes are known to differentially activate various molecules as first-line defense mechanisms including heat shock proteins (HSPs) (Feder and Hofmann, 1999), unfolded protein responses (UPRs), and oxidative stress response pathways to assist cell survival under thermal stress conditions (Alemu et al., 2018; Lin et al., 2019). Additionally, impairments to conventional microtubules and microfilaments due to HS can be correlated to spindle apparatus malfunction and early arrest at metaphase I (MI) (Roth and Hansen, 2005). In order to understand the impact of seasonal thermal stress on follicular function and oocyte physiology, several studies have utilized the *in vitro* exposure of follicular cells (Li et al., 2016; Alemu et al., 2018; Gebremedhn et al., 2020) and oocytes (Al-Katanani et al., 2002; Roth and Hansen, 2004; Roth and Hansen, 2005; Nabenishi et al., 2012; Li et al., 2015; Pavani et al., 2016; Rodrigues et al., 2019; Abdelnour et al., 2020) to elevated incubator temperatures. Accordingly, *in vitro* exposure of oocytes to HS results in impaired developmental potential as evidenced by defective cytoplasmic maturation (Edwards et al., 2005), mitochondrial anomalies (Ferreira et al., 2016), reductions in polar body extrusions (PB) and nuclear maturation (Li et al., 2015; Pavani et al., 2016) as well as increased accumulations of reactive oxygen species (ROS) (Nabenishi et al., 2012), ultimately leading to apoptosis (Nabenishi et al., 2012).

Oocyte growth and maturation occur within the complex follicle microenvironment, involving substantial bi-directional communication between follicular cells (theca, granulosa, and cumulus cells) and the oocyte (Matzuk et al., 2002; Russell et al., 2016). Critical communication and signaling between the varying cell subpopulations during folliculogenesis are paramount to the growth and maturation of developmentally competent oocytes (Matzuk et al., 2002; Alam and Miyano, 2020; Tesfaye et al., 2021). The process by which cells communicate occurs in a multitude of fashions including cytokines (Altan-Bonnet and Mukherjee, 2019), proteins or surface molecules (Ahmed and Xiang, 2011), and gap junctions (Gilula et al., 1978). Of growing interest is cell-to-cell communication *via* membrane-encapsulated vesicles containing surface markers from the cell of origin, harnessed with an abundant amount of bioactive cargoes (RNAs, proteins, lipids, and metabolites, *etc.*). Extracellular vesicles (EVs), an all-inclusive term endorsed by the International Society for Extracellular Vesicles (ISEV), is a term used for a heterogenous population of lipid-enclosed nanoparticles with non-replicative function (Théry et al., 2018). Originally depicted as discarding mechanisms of cellular waste, EVs are growing in popularity as pertinent conveyors of cellular messages *via* their cargo loads, ultimately leading to modification of cellular activity or key properties of target cells (Valadi et al., 2007).

Several studies involving irradiation (Mothersill and Seymour, 1997; Lyng et al., 2000; Chai and Hei, 2008) and thermal stress (Bewicke-Copley et al., 2017) have demonstrated the potential of EVs present in conditioned medium to induce a bystander effect to recipient cells. In those respective studies, naïve cells treated with a medium conditioned by stressed cells showed increased levels of DNA damage and apoptosis when compared to naïve cells treated with a conditioned medium from control cells. Moreover, naïve cells treated with EVs from irradiated or heat-shocked cells have a higher probability to survive subsequent stress, suggesting that EVs can induce adaptive responses. It has also been shown that the supplementation of follicular fluid-derived EVs enhances the developmental capacity of oocytes to reach the blastocyst stage under HS conditions (Rodrigues et al., 2019). In the same study, it was noted that the supplementation of follicular fluid-derived exosomes were superior when compared to the whole follicular fluid, revealing exosomes’ critical role in mitigating HS-induced damage to oocytes. The mechanisms underpinning such bystander effects of EVs are not fully understood, however, it is possible that the changes in protein or RNA contents of EVs may have the capacity to alter the physiology of recipient cells (Bewicke-Copley et al., 2017). MicroRNA profiling of EVs derived from thermally stressed bovine GCs revealed the enrichment of miRNA (miR-1246, miR-2904, and miR-374a), which are known to be involved in pathways associated with cellular response to heat stress, redox status, hypoxia and negative regulation of cell proliferation and inflammatory response (Gebremedhn et al., 2020). In the same study, we demonstrated that the supplementation of thermally stressed EVs to recipient granulosa cells induced tolerance against subsequent thermal stress. Therefore, the current study aimed to test the hypothesis that GC-derived EVs can play a physiologically relevant role in enhancing the survival and viability of bovine oocytes subjected to thermal stress under *in vitro* conditions.

## 2 Materials and methods

### 2.1 Ovary collection and granulosa cell isolation

Local abattoir-derived bovine ovaries were collected and transported *via* an insulated thermos containing physiological saline solution (0.9% NaCl) warmed to 37°C. Upon arrival, ovarian samples were initially washed through a physiological, warmed saline solution and subjected to a 70% ethanol rinse, followed by three repeated washings through a warmed saline solution. Follicular fluid containing granulosa cells and COCs were aspirated from small, growing follicles (3–8 mm in diameter) using a vacuum pump (GenX International; Guelph, ON, Canada) set to approximately 50 mmHg attached to an 18-gauge standard hypodermic needle (Covidien Monoject™; Mansfield, MA, United States) accompanied by a 50 mL sterilized tube (CELLTREAT^®^ Scientific Products; Pepperell, MA, United States). A dense pellet of cellular material containing the COCs was allowed to settle at the bottom of the tube for 10 min, resulting in a visible gradient prior to the collection of cells. The uppermost aqueous portion of the aspirated follicular fluid containing the granulosa cells was then subsequently transferred into a sterile 15 mL tube (Thermo Fisher Scientific; Waltham, MA, United States) with warmed Dulbecco’s Phosphate Buffered Saline without calcium and magnesium chloride (DPBS (×1)) (Sigma-Aldrich; St. Louis, MO, United States).

The upper suspension and warmed DPBS (1X) were mechanically homogenized *via* repetitive pipetting prior to initial centrifugation at 500 xg for 7 min to collect and pelletize the granulosa cells. Next, the supernatant was removed, and the cell pellet was resuspended in red blood cell (RBC) lysis buffer (Sigma-Aldrich; St. Louis, MO, United States) for 3 min. To stop the reaction, the cell pellet was washed with Dulbecco’s Modified Eagle’s Medium/Ham’s Nutrient Mixture F12 (DMEM/F12) (Sigma-Aldrich; St. Louis, MO, United States) supplemented with 10% Exosome-depleted fetal bovine serum (FBS) (System Biosciences; Palo Alto, CA, United States), 1% Penicillin-Streptomycin (Sigma-Aldrich; St. Louis, MO, United States) and 1% Amphotericin B solution (Sigma-Aldrich; St. Louis, MO, United States). Following centrifugation at 500 xg for 5 min, the supernatant was removed, and the cells were washed in DPBS (1X) and resuspended in DMEM/F12 supplemented medium as described above. Cell counts and viability were determined using a hemocytometer (Hausser Scientific; Horsham, PA, United States) and the trypan blue (Sigma-Aldrich; St. Louis, MO, United States) exclusion method.

### 2.2 Granulosa cell culture and exposure to heat stress

Granulosa cells were seeded at 2 × 10^5^ cells per well in 24-well, tissue culture-treated plates (United States Scientific, Inc.; Ocala, FL), suspended in DMEM/F12 supplemented medium as described above. In a humidified atmosphere gauged at 38.5°C containing a 5% CO_2_ environment, cells were cultured for an initial 24 h or until sub-confluent. Following an initial 24 h priming period, the (control) cells continued culture at 38.5°C and the HS cells were transferred to an alternative environment with an elevated thermal temperature of 42°C for the remaining 24 h of the total 48 h culture period. The conditioned spent culture medium was collected and stored at −80°C for EV isolation and downstream application.

### 2.3 Isolation of EVs from spent culture medium

Two replicates of conditioned spent culture medium containing EVs released from cultured granulosa cells were pooled and initially centrifuged at 500 xg for 10 min, followed by 3,000 xg at 4°C to remove residual cellular debris. Following the initial centrifugations, the supernatant containing EVs were then subjected to centrifugation at increased speeds of 17,200 xg for 30 min, followed by filtration through a 0.22 µm sterile syringe filter (Sigma-Aldrich; St. Louis, MO, United States), removing particles larger than 200 nm in diameter including apoptotic bodies. Following this, the purified conditioned spent culture medium was subjected to ultracentrifugation as we previously described in (Saeed-Zidane et al., 2017). Briefly, conditioned spent culture medium from each treatment was subjected to ultracentrifugation at 120,000 xg for 70 min using the Optima XE-90 Ultracentrifuge (Beckman Coulter; Pasadena, CA, United States), in 5.2 mL Ultra-Clear™ Centrifuge Tubes (Beckman Coulter; Pasadena, CA, United States) using the Beckman SW 55Ti rotor (Beckman Coulter; Pasadena, CA, United States). After removing the supernatant, the pelletized EVs were washed using DPBS (1X) and ultracentrifuged under the same conditions as described above. Finally, the supernatant was removed and the EVs were then suspended in 500 µL of DPBS (1X) and stored at −80°C until further analysis. Prior to use, all EV samples were thawed at room temperature to sustain high-yield EV recovery (Trummer et al., 2009; Szatanek et al., 2015).

### 2.4 Morphological and molecular characterization of GC-EVs

Immunoblotting techniques were used to verify the presence of typical EV-specific protein markers (CD63, TSG101, and CD81) in the isolated EVs. Moreover, Cytochrome c (CYCS), a cell-specific marker protein was used to validate the absence of residual cellular debris and cellular protein contaminants in our EV preparations. Full-length blots are provided in (Supplementary Figure S1) and a list of the antibodies used is indicated in (Supplementary Table S1).

Characterizing the size and concentration of EV samples were performed using nanoparticle tracking analysis (NTA). Briefly, 5 µL of purified EVs were diluted in 995 µL of DPBS (1X) and assembled into the ZetaView^®^ QUATT 4 Nanosight Instrument (Particle Metrix; Ammersee, Germany) fitted with four respective lasers (405/488/520/640 nm sources). For each sample, video measurements were recorded, and 11 positions were analyzed using the Software Zetaview (version 8.05.12 SP1). Similarly, the morphology and size of EVs were determined using a particle beam of electrons for visualization using transmission electron microscopy (TEM). For this, a small volume of isolated EV samples (6–8 µL) was applied to a carbon-coated, copper mesh TEM grid for 1–2 min, followed by negative staining with 2% aqueous uranyl acetate. TEM imaging was done on an FEI/TFS Tecnai T12 Spirit TEM (FEI Company; Hillsboro, OR, United States), operating at 100 kV, with an AMT CCD. We have submitted all relevant data from our experiments to the EV-TRACK knowledgebase (EV-TRACK ID: EV220412) (van Deun et al., 2017).

### 2.5 Fluorescence labeling of GC-EVs and assessing cellular uptake

EVs were labeled with PKH26 (Mini26-1KT; Sigma-Aldrich; St. Louis, MO, United States), a red fluorescent linker dye responsible for labeling lipid membranes as described in (Da Silveira et al., 2012). Briefly, EVs (3.5 × 10^11^ particles/mL) were incubated with 1 µL of PKH26 (4 × 10^-6^ M) for 5 min at room temperature in the dark, followed by immediate quenching with 1% BSA (A6003; Sigma-Aldrich; St. Louis, MO, United States) for 1 min. Samples were precipitated using Exo-spin™ buffer-exosome precipitation reagent (CellGS^®^; St. Louis. MO, United States) for 60 min in the dark at 4°C, then centrifuged at 16,000 xg for 30 min and resuspended in 100 μL of DPBS (1X) before purification using the Exo-spin™ columns according to the manufacturer’s protocol. As a negative control, DPBS (1X) was incubated with PKH26 and treated in the same manner as described above. Labeled EVs or DPBS (1X) (negative control) were used to prove EV uptake by CCs surrounding intact COCs as detailed below.

COCs obtained from follicles ranging in size from 3 to 8 mm in diameter were aspirated from abattoir-derived ovaries. Three COCs were cultured in 20 µL micro drops offset with 20% v/v of GC-EVs suspended in DPBS (1X) measured at 0.3 mg/mL of total protein under oil for a 23 ± 1 h exposure length. Following incubation, intact COCs were washed in CSU chemically defined medium for the handling of oocytes (HCDM-M) (De La Torre-Sanchez, Jose Fernando et al., 2006), fixed with 4% paraformaldehyde, mounted to a poly-L-lysine coated slide (Newcomer Supply, Inc.; Middleton, WI, United States), and allowed to dry for 24 h at 4°C in the dark. Visualization of fluorescently-labeled EVs within the CCs surrounding the oocyte was ascertained using confocal microscopy (Olympus Fluoview FV10-ASW 4.1) (Olympus; Tokyo, Japan) after excitation of COCs at 559 nm with a ×60 objective lens. Labeled DPBS (1X) was used as negative control within this experiment.

### 2.6 *In vitro* maturation and exposure to heat stress

The collection of COCs from slaughterhouse ovaries was done as described above. Once COCs were collected, groups of 50 oocytes were transferred into individual wells, inside 4-well culture dishes (Thermo Fisher Scientific; Waltham, MA, United States) containing 1 mL of CSU chemically defined medium for *in vitro* maturation of oocytes (IVM) pre-equilibrated at 38.5°C in 5% CO_2_ (De La Torre-Sanchez, Jose Fernando et al., 2006). At the time IVM medium were set to equilibrate, all mediums irrespective of downstream application were supplemented with 15 ng/mL NIDDK-oFSH-20, 1 μg/mL USDA-LH-B-5, 1 μg/mL estradiol 17β, 50 ng/μL epidermal growth factor, and 0.1 mM cysteamine (De La Torre-Sanchez, Jose Fernando et al., 2006).

For experiments with heat stress, treatments were arranged in a 2 × 4 factorial design with primary effects of incubation temperature during maturation (38.5°C vs. 41°C) and with four culture mediums: non-supplemented control (NC), vehicle [DPBS (1X)], EVs derived from granulosa cells subjected to the normal physiological body temperature of the cow (38.5°C) (normal EVs: N-EVs) or EVs derived from granulosa cells subjected to thermal stress (42°C) (stressed EVs: S-EVs). In brief, COCs were incubated with 20% v/v of DPBS (1X), and treatments with GC-EVs were offset with equal concentrations of total protein (0.3 mg/mL) assessed using a NanoDrop 2000 Spectrophotometer (Thermo Scientific; Waltham, MA, United States), selected based on previous literature (Almiñana et al., 2017; Alcântara-Neto et al., 2020a; Alcântara-Neto et al., 2020b; Abumaghaid et al., 2022; Mateo-Otero et al., 2022). For all experimental groups, control COCs matured at a temperature of 38.5°C throughout the maturation period (23 ± 1 h), while thermally stressed COCs were subjected to an initial 8 h priming period at 38.5°C and then transferred to 41°C in an atmosphere of 5% CO_2_ in humidified air for the remaining 15 ± 1 h of the 23 ± 1 h maturation period.

### 2.7 *In vitro* fertilization and embryo culture

After maturation, COCs were transferred to 4-well culture dishes containing 430 µL of equilibrated CSU chemically defined medium for *in vitro* fertilization (F-CDM/well) (De La Torre-Sanchez, Jose Fernando et al., 2006). In a minimal volume of maturation medium, COCs were transferred into F-CDM and held at 38.5°C and 5% CO_2_ in humidified air until IVF was completed using bull sperm generated from a single ejaculation. Frozen-thawed semen were subjected to a multi-layer, 45%/90% Percoll^®^ (Sigma-Aldrich; St. Louis, MO, United States) gradient and centrifuged at 800 xg for 20 min to separate viable, motile sperm (Parrish et al., 1995). Following this, the supernatant was removed and the remaining sperm pellet was washed with 2 mL of CSU chemically defined medium for the handling of early embryos (HCDM-1) and centrifuged at 300 xg for 5 min (De La Torre-Sanchez, Jose Fernando et al., 2006). The concentration of spermatozoa was determined using a hemocytometer and adjusted to 5 × 10^6^ sperm/mL using equilibrated F-CDM. Finally, sperm were added to COCs at a concentration of 0.5 × 10^6^ sperm/mL, and co-incubated for 18 h at 38.5°C in 5% CO_2_ in humidified air.

Following exposure with spermatozoa, presumptive zygotes were vortexed at maximum speed for 60–90 s to slough remaining CCs. Denuded zygotes were then transferred through a series of HCDM-1 wash drops, removing residual cells and spermatozoa (De La Torre-Sanchez, Jose Fernando et al., 2006). Zygotes rid of excess cellular debris were then transferred (in groups of 50) to pre-equilibrated 4-well dishes containing 500 µL of CSU chemically defined medium for *in vitro* culture of early embryos (CDM-1) and cultured for 56 h in a tri-gas incubator regulated to 38.5°C in 5% CO_2_, 5% O_2,_ and 90% N_2_. Following an initial culture of 56 h, development was assessed by embryo cleavage in warmed micro drops of CSU chemically defined medium for the handling of late embryos (HCDM-2) (De La Torre-Sanchez, Jose Fernando et al., 2006). Those embryos with ≥2 blastomeres were transferred (in groups ≤35 embryos) to pre-equilibrated 4-well dishes containing 500 µL of CSU chemically defined medium for *in vitro* culture of late embryos (CDM-2) for an additional 120 h of culture in a humidified chamber at 38.5°C in 5% CO_2_, 5% O_2_ and 90% N_2_ (De La Torre-Sanchez, Jose Fernando et al., 2006). Blastocyst rates were determined on day 7 (168 h) and day 8 (192 h) post-onset of IVF. Blastocysts were evaluated according to their respective stage of development and graded morphologically in accordance with the IETS guidelines (Robertson, 1998).

### 2.8 Quantitative analysis of cumulus cell expansion

To determine the effect of EV supplementation on cumulus expansion, arbitrary lines were drawn to measure the diameter of single cumulus-oocyte complexes (four replicates; a total of 113–252 COCs per treatment) using the Olympus Cellsens software (Olympus; Tokyo, Japan). Measurements were ascertained from groups of COCs and the mean diameter before maturation was subtracted from the mean diameter after maturation to determine the mean cumulus expansion in µm’s. The diameter of COCs was measured before and after maturation at differing ambient temperatures from recorded images (.jpg) using an inverted microscope (IX73; Olympus; Tokyo, Japan).

### 2.9 Total RNA isolation and quality control

Total RNA was isolated from three replicates of CCs (each pool contains CCs derived from 50 COCs) using the miRNeasy^®^ mini kit (Qiagen; Hilden, Germany), and on-column DNA digestion was performed using RNase-free DNase (Qiagen; Hilden, Germany). Moreover, total RNA was extracted from three replicate pools of denuded oocytes following the completion of *in vitro* maturation (each pool consisting of 50 oocytes) and four replicate pools of blastocysts (each pool containing 5 blastocysts) using the RNeasy^®^ plus micro kit (Qiagen; Hilden, Germany). Total RNA sample concentration and integrity were assessed using a NanoDrop 2000 Spectrophotometer (Thermo Scientific; Waltham, MA, United States). RNA samples were stored at −80°C until further use.

### 2.10 Quantification of cumulus expansion and stress-associated genes using qRT-PCR

The relative abundance of selected cumulus expansion marker genes (PTGS2, EGFR, and PTX3) were quantified in CCs isolated from matured COCs (three replicates; CCs from a pool of 50 COCs per replicate) using qRT-PCR. Cumulus cells were collected immediately following *in vitro* maturation by recurrent pipetting through a narrow pipette, followed by vortexing and centrifugation. Complete removal of CCs from the oocytes was assessed under a stereo microscope (SZX16; Olympus; Tokyo, Japan) and snap frozen in liquid nitrogen then stored at −80°C until further analysis. For the analysis, synthesized cDNA was reverse transcribed from equal amounts of total RNA (85 ng) using the SuperScript™ III First-Strand Synthesis Super Mix Kit (Invitrogen; Carlsbad, CA, United States) with random hexamer and oligo d(T) 20 primers. Similarly, the transcript abundance of selected stress-related genes (NRF2, SOD1, HSP70, HSP90, GRP78, and GRP94) were quantified in CCs (three replicates; CCs from a pool of 50 COCs per replicate), *in vitro* matured denuded oocytes (three replicates; a pool of 50 COCs per replicate) and blastocysts (four replicates; a pool of 5 blastocysts per replicate) using qRT-PCR. For this, cDNA was obtained after reverse transcription of equal amounts of total RNA (85 ng from CCs, 31 ng from oocytes, and 13 ng from blastocysts) as described above. The geometric mean of expression levels of β-ACTIN and GAPDH was used to normalize the expression of candidate genes. Gene-specific primers were designed using Primer-Blast (https://www.ncbi.nlm.nih.gov/tools/primer-blast/) and the list of primers is indicated in Supplementary Table S2. qRT-PCR analysis were performed using the CFX96 Touch Real-Time PCR Detection System (Bio-Rad; Hercules, CA, United States) and data was analyzed using the comparative C(T) method (Schmittgen and Livak, 2008).

### 2.11 Measurement of intracellular ROS accumulation

The intracellular ROS accumulation in oocytes (three replicates; a total of 15–50 oocytes per treatment) and blastocysts (three replicates; a total of 6-9 blastocysts per treatment) from the various treatment groups was determined as we described previously (Gebremedhn et al., 2020). Briefly, oocytes (*n* = 32 on average) and embryos (*n* = 8 on average) from each treatment group were washed twice in phosphate-buffered saline-polyvinylpyrrolidone DPBS (1X)-PVP (0.1%) (P0930; Sigma-Aldrich; St. Louis, MO, United States), placed in 50 µL droplets of 10 µM 2ʹ,7ʹ-Dichlorofluorescin Diacetate (H₂DCFDA) dissolved in H-CDMM or H-2 (oocyte or blastocyst) (287,810; Sigma-Aldrich; St. Louis, MO, United States), and incubated at 38.5°C in the respective culture environment suitable for their specific stage of development for 30 min. After incubation, the oocytes and embryos were washed three times in DPBS (1X)-PVP (0.1%) and then observed under an inverted fluorescent microscope (IX73; Olympus; Tokyo, Japan) using ×20 magnification. The fluorescence of individual specimens was measured from.jpg images and analyzed using ImageJ software (National Institute of Health; Bethesda, MD, United States) after normalization through subtraction of the background intensity. All images were captured under the same parameters for exposure time and light intensity to avoid fluorescent saturation using a green fluorescent filter with an excitation wavelength of 494 nm and an emission wavelength of 518 nm. Results are plotted as the relative intensity of fluorescence.

### 2.12 Analysis of mitochondrial membrane potential (MMP)

MMP (**∆**Ψ_**m**_) in oocytes (duplicates; a total of 12-22 oocytes per treatment) and blastocysts (duplicates; a total of 7-11 blastocysts per treatment) were analyzed by staining with 5,5′,6,6′-tetrachloro-1,1′,3,3′tetraethylbenzimidazolylcarbocynanine iodide (JC-1, Invitrogen; Carlsbad, CA, United States) dissolved in H-CDMM or H-2 (oocyte or blastocyst), as an indicator of mitochondrial activity, as previously described (Pasquariello et al., 2019). Briefly, denuded matured oocytes and blastocysts from the various treatment groups were stained using 2 µM JC-1 for 30 min in the dark at 38.5°C, in the optimal culture environment for their respective stage and development to express two types of fluorescence. Oocytes and blastocysts were then washed three times in DPBS (1X)-PVP (0.1%) and fluorescent images were taken immediately using an inverted laser scanning confocal microscope (ZEISS LSM980, Carl Zeiss Microscopy, Jena, Germany) using a ×20 magnification air objective. Fluorescent images were ascertained using the lowest laser power to give a sufficient signal-to-noise ratio to avoid saturation of the fluorophore. Physiological temperature (37°C) and CO_2_ level (5%) were maintained inside the incubation chamber amidst imaging. Imaging was performed using a wavelength of 488 nm for J-monomers and excitation/emission at 490/525 nm (FITC filter/green fluorescence) indicating low membrane potential. Similarly, images for J-aggregates were carried out using a wavelength of 561 nm and excitation/emission at 596/615 nm (TRITC filter/red fluorescence) indicating high membrane potential. Subsequently, the ratio of red to green fluorescence was used to determine mitochondrial membrane potential, expressed as an arbitrary unit as previously described (Silva et al., 2015; Pasquariello et al., 2019). All czi. images were analyzed using ImageJ software (National Institute of Health; Bethesda, MD, United States).

### 2.13 Fluorescent nuclear staining and terminal deoxynucleotidyl transferase-mediated dUTP nick-end labeling (TUNEL) assay

Blastocyst-stage embryos from the different treatment groups (three replicates; a total of 18-30 blastocysts per treatment) were assessed for proliferative capacity based on total cell counts using a protocol adapted from (Critser and First, 1986) with some modifications. Briefly, blastocysts were washed three times in 100 µL drops of phosphate buffered saline-polyvinylalcohol DPBS (1X)-PVA (0.1%) (P8136; Sigma-Aldrich; St. Louis, MO, United States), for 5 min each time. Blastocysts were then immediately transferred into 1.5 μg/mL Hoechst 33,342 solution (H3570; Thermo Fisher Scientific; Waltham, MA, United States) and stained for 10–15 min at room temperature in the dark. Embryos were then washed as previously described and mounted on a poly-L-lysine coated slide (Newcomer Supply, Inc.; Middleton, WI, United States) with 5–10 µL ProLong Diamond Antifade Mountant (P36966; Invitrogen; Carlsbad, CA, United States). Alternatively, cellular DNA fragmentation in blastocysts from the various treatment groups (three replicates; a total of 19-41 blastocysts per treatment) was determined using an *in situ* apoptotic cell detection kit, DeadEnd™ Fluorometric TUNEL System according to the manufacturer’s instruction (TB235; Promega; Madison, WI, United States). Briefly, blastocysts were fixed in 4% paraformaldehyde (PFA) for 25 min at 4°C. Following fixing, blastocysts were washed twice in 0.1% Tween/PVP in DPBS (1X) (Bio-Rad; Hercules, CA, United States; P0930; Sigma-Aldrich; St. Louis, MO, United States) and permeabilized in 0.3% Triton X-100 in DPBS (1X) (X100-100ML; Sigma-Aldrich; St. Louis, MO, United States) for 5 min. After permeabilization, blastocysts were washed as detailed previously and incubated in equilibration buffer prior to labeling with the TUNEL assay kit cocktail at 38.5°C for 60 min in a dark humidified chamber as suggested in the manufacturer’s protocol. To stop the reaction, blastocysts were moved to 2X SSC for 15 min and washed an additional three times before mounting on a poly-L-lysine coated slide (Newcomer Supply, Inc.; Middleton, WI, United States) with 5–10 µL ProLong Diamond Antifade Mountant (P36966; Invitrogen; Carlsbad, CA, United States) and a coverslip, allowing to dry flat (>24 h). Hoechst/DAPI-labeled or TUNEL-positive nuclei were observed under an inverted fluorescent microscope (IX73; Olympus; Tokyo, Japan) using ×20 magnification, under the same parameters using fitted blue (345/455) and green (494/518) fluorescent filters. For the analysis, fluorescent images were converted to grayscale to accurately adjust what is fluorescently stained (elements in the foreground, as well as elements in the background). Subsequently, the fluorescently labeled cells were then filled appropriately to determine the borders of individual nuclei based on relative size for counting purposes irrespective of the fluorophore utilized (blue/green).

### 2.14 Experimental design

A schematic overview of the experimental design is shown in (Supplementary Figure S2). Following the generation of GC-EV subsets (N-EVs and S-EVs) for isolation and characterization, their subsequent effect was evaluated on COCs cultured under normal (38.5°C) and HS (41°C) conditions during IVM. For this purpose, COCs were collected by aspirating small-growing follicles (3–8 mm in diameter) from abattoir-derived reproductive ovaries. To avoid result bias, Grade 1 and Grade 2 COCs were selected for further functional studies and placed into 4-well culture dishes containing four distinct equilibrated mediums: non-supplemented control (NC), vehicle [DPBS (1X)], normal EVs (N-EVs) or stressed EVs (S-EVs) suspeneded in 20% v/v of DPBS (1X), with GC-EV treatments offset with equal concentrations of total protein (0.3mg/ml). Concluding IVM, *in vitro* matured oocytes were assessed for EV uptake and cumulus expansion. Additionally, selected stress-associated transcripts were investigated among resected cumulus cells and denuded matured oocytes, and confocal microscopy was utilized to determine ROS production and mitochondrial activity among *in vitro* matured oocytes. In parallel, aside from experiments conducted immediately following IVM, subsequent IVF cycles were carried out to investigate the downstream effects of GC-EV supplementation during oocyte maturation on the resulting blastocyst development. Developmental competence rates were collected, and blastocysts were similarly evaluated for selected stress-associated transcript abundance and dually stained for ROS accumulation, total cell counts, apoptotic indexes, and mitochondrial membrane potential as a means to monitor the quality of the produced blastocysts.

### 2.15 Statistical analysis

Data were analyzed in GraphPad Prism version 8.4.2 (GraphPad; San Diego, CA, United States). Statistical differences between the mean values of more than two groups under the varying culture conditions were compared using Two-way Analysis of Variance (ANOVA) followed by Tukey’s Multiple Comparisons Tests. Alternatively, statistical differences amid gene expression datasets were assessed between the mean values of more than two groups under the same culture conditions using One-way Analysis of Variance (ANOVA) followed by Tukey’s Multiple Comparisons Tests. Data are presented as the Mean ± SEM of biological replicates. Statistical significance was identified at *p* ≤ 0.05.

## 3 Results

### 3.1 Characterization of GC-EVs released into the conditioned medium from *in vitro* cultured follicular GCs

**FIGURE 1:**
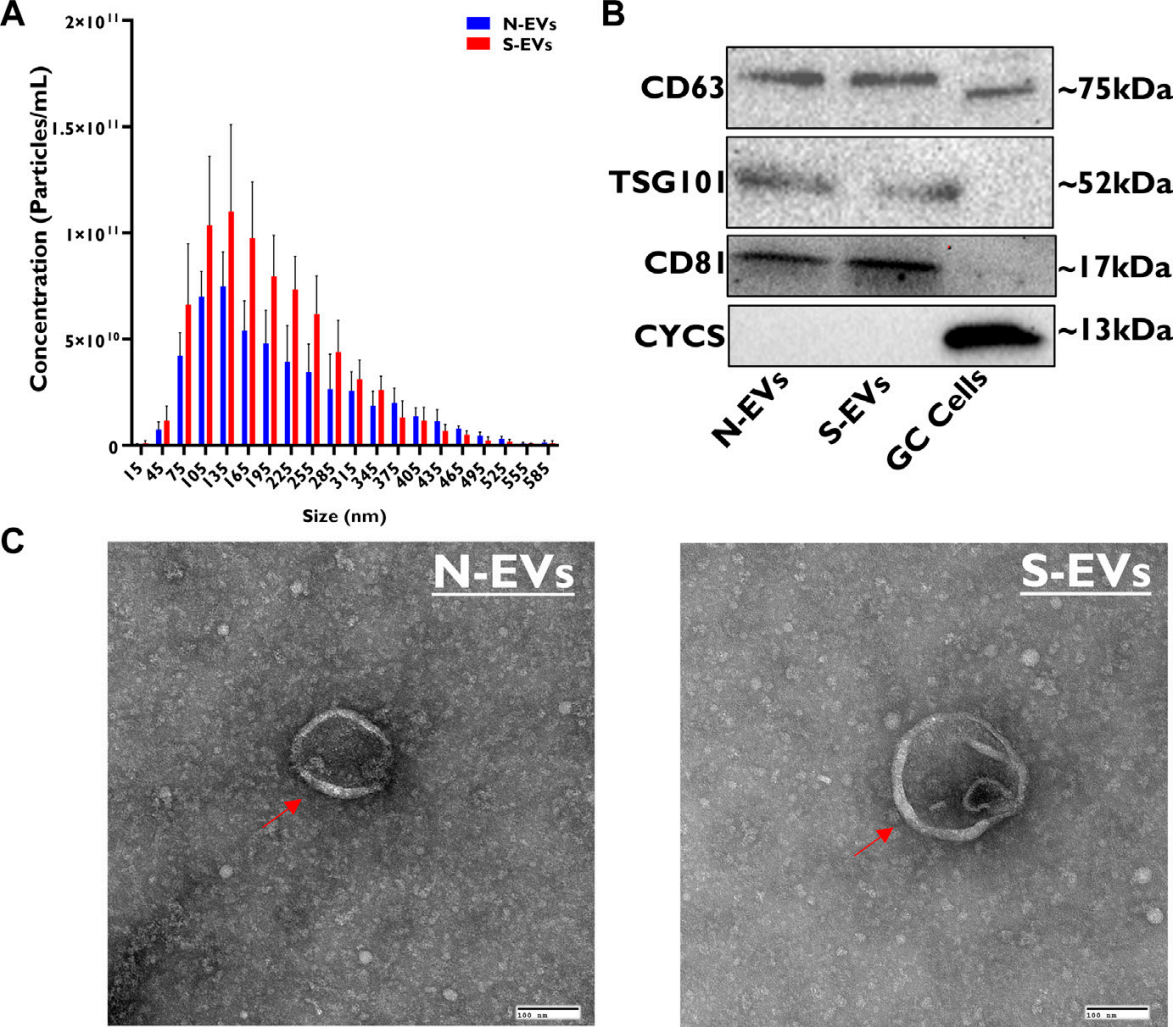
Morphological and molecular characterization of GC-EVs. The relative size of EVs isolated from cultured granulosa cells spent culture medium was determined using nanoparticle tracking analysis (NTA). The median size of N-EVs was 137.75 nm (blue) compared to S-EVs 147.95 nm (red). Granulosa cells subjected to thermal stress released a significantly higher quantity of particles (red) and had tendencies to be larger in size (nm) compared to those subjected to normal ambient temperatures (blue) **(A)**. Immunoblotting analysis for EV-specific protein markers (CD63, TSG101, and CD81) and cellular-specific protein marker (CYCS) were verified to determine protein lysate composition in N-EVs, S-EVs, and GCs as a positive control. Full-length blots are presented in Supplementary Figure S1 **(B)**. Transmission electron microscopy (TEM) representative images from N-EVs and S-EVs (marked with red arrows) show the morphology of the lipid bilayer membrane of EV particles. Scale bar, 100 nm **(C)**.

Vesicle size and concentration differences between N-EVs and S-EVs were assessed using Nanoparticle Tracking Analysis (NTA). The concentration of N-EVs and S-EVs were 6.70E+11 ± 3.50E+11 and 1.00E+12 ± 5.10E+11 particles/mL, with median sizes of 137.75 and 147.95 nm, respectively. Exposure to heat stress resulted in the release of a significantly higher quantity of EVs with a tendency of having larger size particles (Figure 1A). Western blotting analysis of N-EVs and S-EVs revealed positive bands for EV- specific transmembrane proteins (CD63, TSG101, and CD81), simultaneously showing negative results for mitochondrial protein marker (CYCS) (Figure 1B). Similarly, TEM imaging showed a clear indication that vesicles are encapsulated with the visible appearance of a bilipid membrane (Figure 1C).

### 3.2 GC-EVs shuttle protective messages against heat stress to enhance the thermotolerance of bovine cumulus-oocyte complexes

**FIGURE 2:**
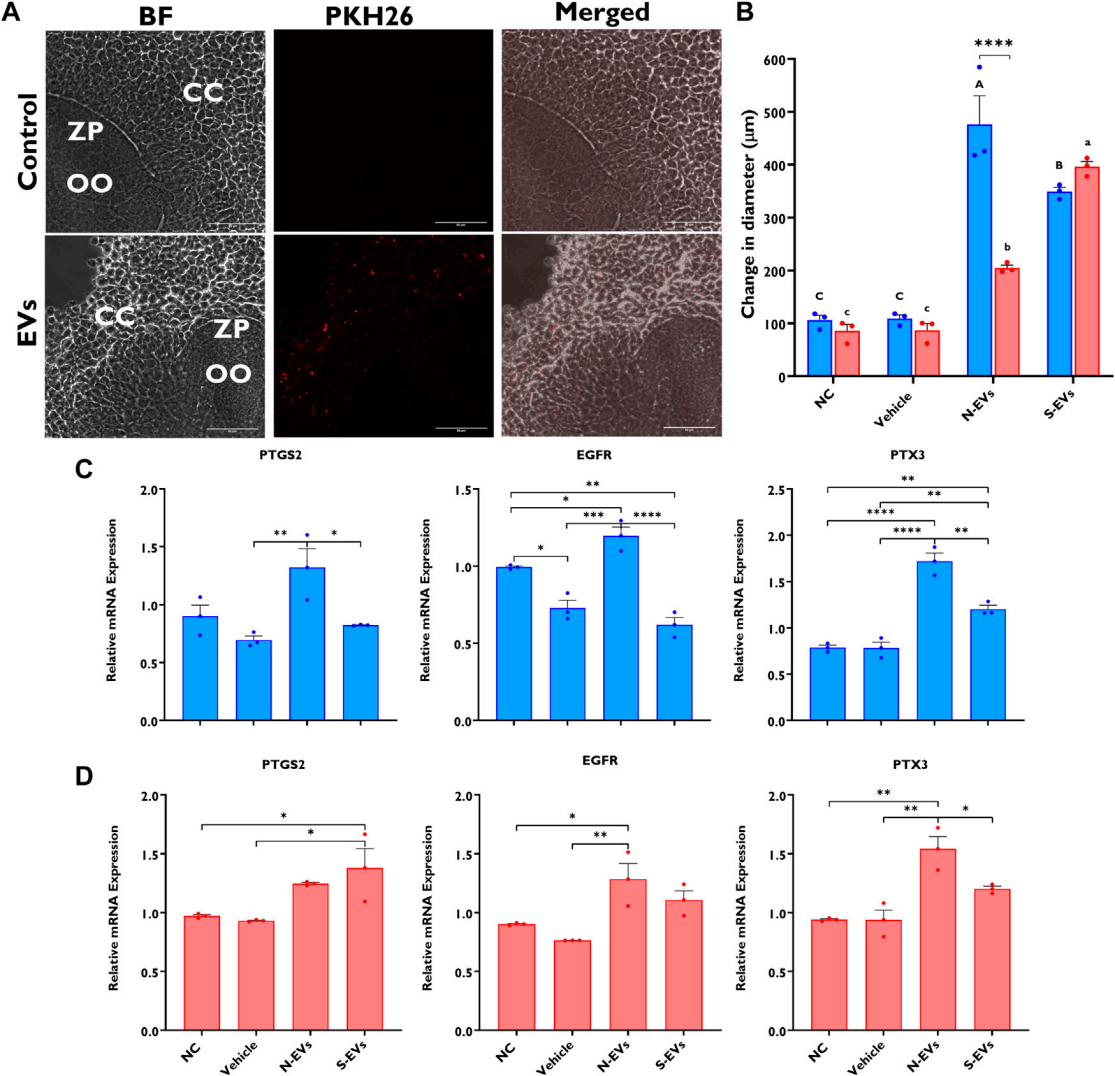
Effects of GC-EV supplementation on receptivity of follicular cells EV uptake following maturation at 38.5°C (blue) and 41°C (red) and cumulus expansion. Confocal images of intact cumulus-oocyte complexes co-incubated with PKH26 labeled GC-EVs for 24 h during in vitro maturation. Scale bar, 50 µM **(A)**. Follicular cell uptake of PKH26 labeled EVs is shown in the surrounding cumulus cells (CC), in relation to the oocyte (OO) and respective zona pellucida (ZP). Measurements for cumulus expansion were obtained before and after maturation using the Olympus Cellsens software. The increase in diameter was determined by subtracting the mean diameter before maturation from the mean diameter after maturation **(B)**. Results were carried out in four replicates (a total of 113-252 COCs per treatment). Data among treatments represent the mean±SEM and the differences between means were analyzed using Two-way ANOVA followed by Tukey’s Multiple Comparisons Test. Bars with different letters (38.5°C; uppercase) (41°C; lowercase) indicate statistically significant differences of at least (*p* < 0.05) between treatments under the same culture conditions, while (*) indicate statistical differences between the same treatment under different culture environments during IVM (****p < 0.0001). The expression of cumulus expansion marker genes was assessed using qRT-PCR from cumulus cells isolated from oocytes in triplicates (a total of 50 COCs per treatment) cultured under thermoneutral **(C)** and heat stress **(D)** conditions. Data among treatments cultured under the same environment represent the mean ± SEM and the differences between means were analyzed using One-way ANOVA followed by Tukey’s Multiple Comparisons Test. (*p < 0.05), (**p < 0.01), (***p < 0.001), (****p < 0.0001).

As a means to validate EVs’ capacity to mediate communicative interactions supporting oocyte and embryo development, we confirmed GC-EV uptake by COCs using fluorescence microscopy. After 24 h co-incubation of labeled EVs with COCs, we detected PKH-26 positive EVs in the CCs surrounding the oocyte, but not in the oocyte cytoplasm (Figure 2A). Subsequent investigation of the impact of EV supplementation during oocyte maturation revealed that both N-EVs and S-EVs had positive impacts on cumulus expansion compared to non-supplemented controls and vehicle-supplemented groups, irrespective of the presence or absence of thermal stress exposure. Interestingly, under normal physiological temperature (38.5°C), N-EVs significantly increased cumulus expansion compared to the NC (*p* < 0.0001) and vehicle (*p* < 0.0001). Similar results were found for COCs supplemented with S-EVs during oocyte maturation and cultured under HS conditions when compared to the NC (*p* < 0.0001) and vehicle (*p* < 0.0001) (Figure 2B). We further validated differential expression patterns of cumulus expansion marker genes in the CCs from oocytes of the various treatment groups. Results showed that the expression patterns of PTGS2, EGFR, and PTX3 were higher within the CCs from COCs supplemented with N-EVs cultured both under normal and thermal stress conditions. Under HS conditions, N-EVs and S-EVs tended to act cohesively with both expression patterns being upregulated when compared to the NC and vehicle (Figures 2C, D). Collectively our data indicate significant differences between GC-EV supplemented treatments (*p* < 0.0001), culture temperatures (*p* = 0.0015), as well as an interaction between temperature and treatment (*p* = 0.0002).

**FIGURE 3:**
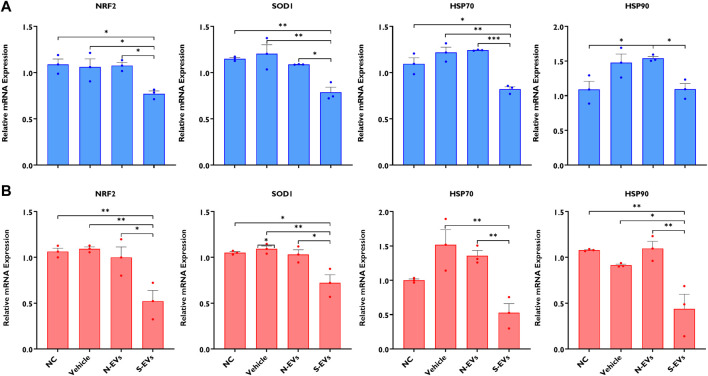
The impact of GC-EV supplementation on transcript levels of stress-associated genes in cumulus cells from cumulus-oocyte complexes following maturation at 38.5°C (blue) and 41°C (red)**.** The expression of stress-associated genes was significantly diminished in cumulus cells from oocytes supplemented with S-EVs and matured at 38.5°C **(A)** and 41°C **(B)**. Results are from cumulus cells removed from a total of three pools of 50 oocytes per pool for each treatment. Data represent the mean ± SEM and the differences between means were analyzed using One-way ANOVA followed by Tukey’s Multiple Comparisons Test. (*p < 0.05), (**p < 0.01), (***p < 0.001).

**FIGURE 4:**
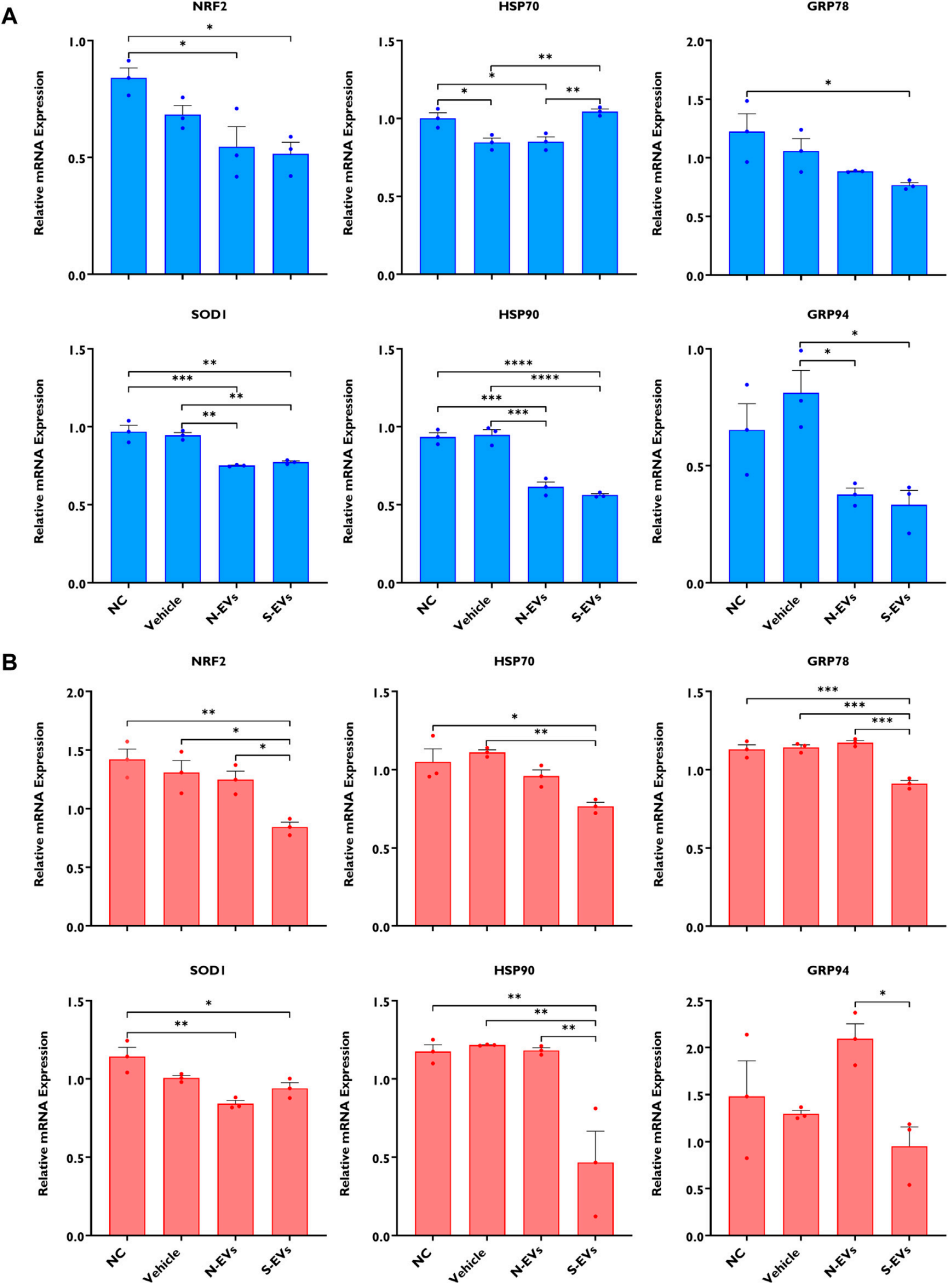
The impact of GC-EV supplementation on transcript levels of stress-associated genes in oocytes following maturation at 38.5°C (blue) and 41°C (red). The expression of stress-associated genes were significantly diminished in oocytes supplemented with N-EVs and cultured at 38.5°C **(A)**, and similar decreases in gene expression were visualized in oocytes supplemented with S-EVs and exposed to HS **(B)**. Results are from a total of three pools of 50 oocytes per pool for each treatment. Data represent the mean ± SEM and the differences between means were analyzed using One-way ANOVA followed by Tukey’s Multiple Comparisons Test. (*p < 0.05), (**p < 0.01), (***p < 0.001), (****p < 0.0001).

The supplementation of S-EVs to COCs at the time of maturation significantly decreased the relative expression of NRF2, SOD1, HSP70, and HSP90 in CCs from COCs, under both ambient temperatures alike (Figures 3A, B). Contrasting evidence showed that N-EVs did not show measurable differences in the suppression of stress-associated transcripts compared to the NC and vehicle under both normal physiological temperature and when subjected to HS (Figures 3A, B). As shown in Figure 4, the supplementation of both N-EVs and S-EVs resulted in the suppression of the majority of stress-associated genes in oocytes cultured either under normal or thermal stress conditions. Contrary to these findings, *HSP70* was significantly enriched within the oocytes supplemented with S-EVs and cultured at 38.5°C (Figure 4A), however, significantly suppressed under thermal stress conditions (Figure 4B).

### 3.3 GC-EVs prevent ROS accumulation and promote mitochondrial function in oocytes under heat-stress conditions

**FIGURE 5:**
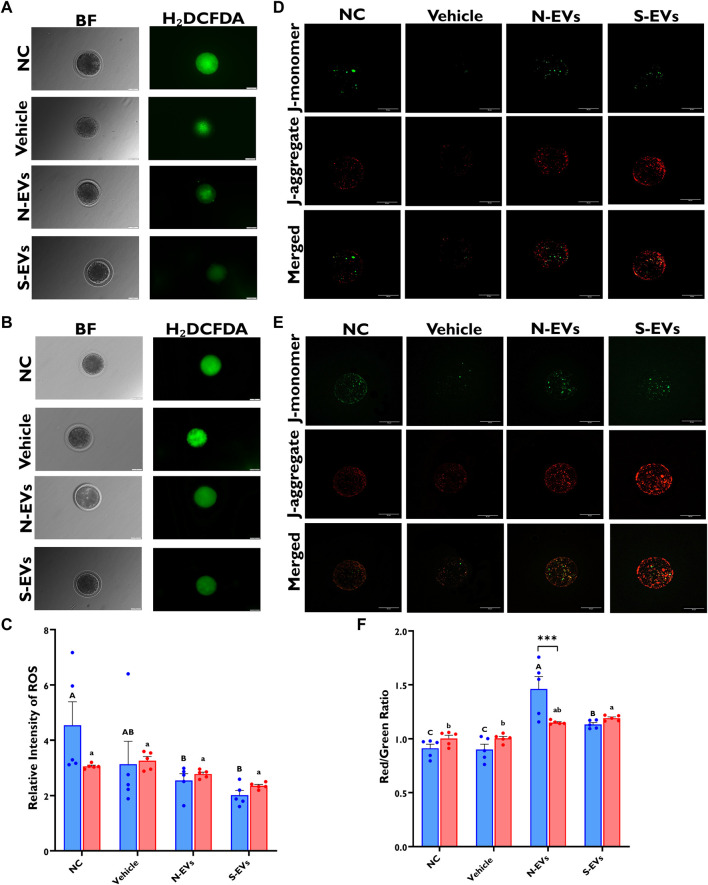
The beneficial effects of GC-EVs on oocyte function under *in vitro* normal physiological (38.5°C; blue) and HS (41°C; red) conditions. Fluorescence images of *in vitro* matured bovine oocytes treated with 2ʹ,7ʹ-Dichlorofluorescin Diacetate (H₂DCFDA) were captured for the measurement of intracellular ROS levels in single oocytes. Scale bar, 50 µM [38.5°C; **(A)**] [41°C; **(B)**]. Quantification of the relative fluorescence intensity in single oocytes were carried out in triplicates (total = of 15–50 oocytes per treatment) **(C)**. Fluorescence images of *in vitro* matured bovine oocytes treated with 5,5′,6,6′-tetrachloro-1,1′,3,3′tetraethylbenzimidazolylcarbocynanine iodide (JC-1) for the measurement of MMP (∆Ψm), an indicator of mitochondrial activity in single oocytes. Scale bar, 50 µM [38.5°C; **(D)**] [41°C; **(E)**]. Quantification of the relative fluorescence intensity (red/green ratio) in single oocytes were carried out in duplicates (total = of 12–22 oocytes per treatment) **(F)**. Data among treatments represent the mean ± SEM and the differences between means were analyzed using Two-way ANOVA followed by Tukey’s Multiple Comparisons Test. Bars with different letters (38.5°C; uppercase) (41°C; lowercase) indicate statistically significant differences of at least (*p* < 0.05) between treatments under the same culture conditions, while (*) indicate statistical differences between the same treatment under different culture environments during IVM (***p < 0.001).

ROS production and mitochondrial membrane potential were investigated in *in vitro* matured oocytes supplemented with GC-derived EVs *via* fluorescence microscopy. Diminished fluorescent signals of ROS were seen in those oocytes supplemented with S-EVs and matured at 41°C, compared to the other groups (Figures 5B, C). Similarly, under normal ambient temperature, fluorescent ROS signals were substantially reduced in oocytes supplemented with N-EVs and S-EVs when compared to controls (Figures 5A, C). Our data also concur significant differences between GC-EV supplemented treatments (*p* = 0.0208), albeit no measurable differences among temperatures or temperature and treatment interactions. Subsequent investigation of the mitochondrial membrane potential in oocytes by JC-1 staining revealed that under HS conditions, oocytes supplemented with both N-EVs and S-EVs showed markedly high mitochondrial membrane potential (∆Ψ_m_) (Figures 5E, F). However, under normal physiological conditions, the highest ∆Ψ_m_ were observed in the treatment group supplemented with N-EVs (Figures 5D, F). Statistical comparisons for JC-1 indicated, significant differences between GC-EV supplemented treatments (*p* < 0.0001), however, no statistical differences among culture temperatures, amid a significant interaction between culture temperature and GC-EV supplemented treatments (*p* = 0.0002).

### 3.4 The effect of GC-EV supplementation during oocyte maturation on developmental competence of oocytes post IVF

**FIGURE 6:**
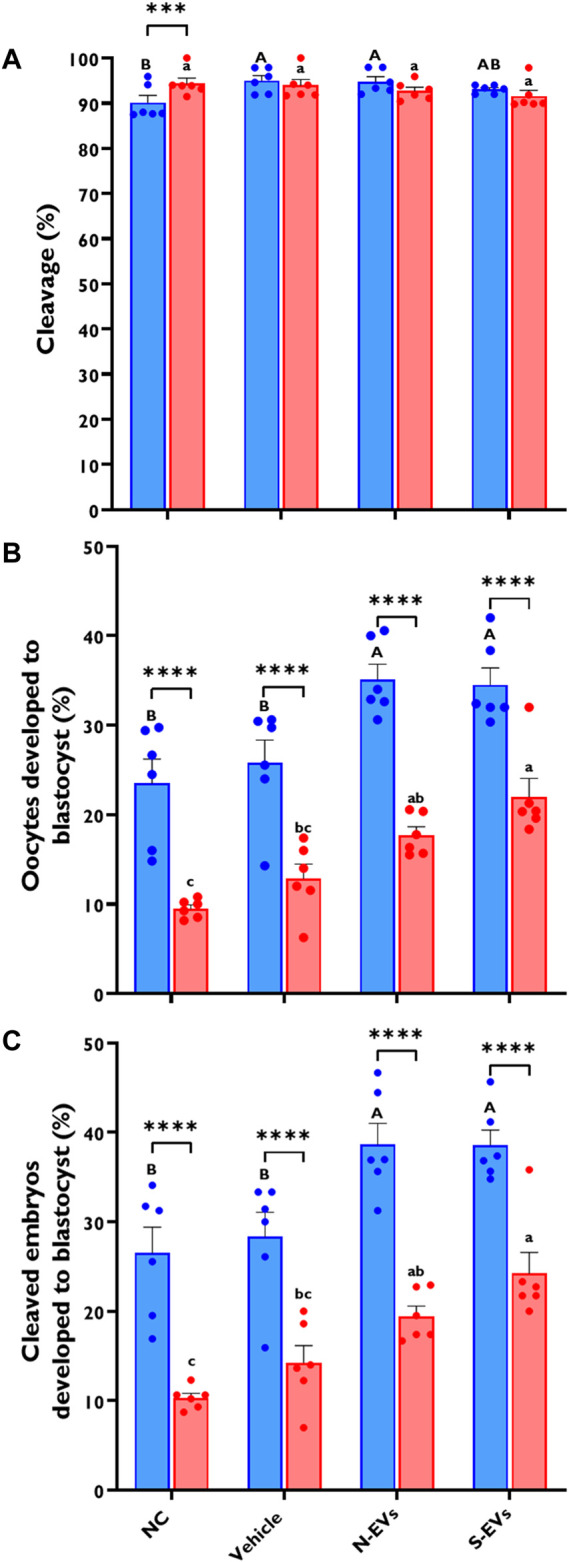
The effect of GC-EVs on the developmental capacity of oocytes matured at 38.5°C (blue) and 41°C (red). Results are from a total of six replicates (281–299 total oocytes per treatment). Oocytes supplemented with N-EVs and cultured under 38.5°C showed significant improvements in cleavage percentage compared to the NC, while no visual differences were observed among treatments under HS conditions **(A)**. The percentage of oocytes developed into blastocysts was significantly affected by maturation temperature with significant increases in developmental competence in those treatments supplemented with GC-EVs **(B)**. Similarly, the percentage of cleaved embryos developed into blastocysts was linearly affected by maturation temperature with significant protection from inherent stress visualized in those treatments supplemented with GC-EVs **(C)**. Data among treatments represent the mean ± SEM and the differences between means were analyzed using Two-way ANOVA followed by Tukey’s Multiple Comparisons Test. Bars with different letters (38.5°C; uppercase) (41°C; lowercase) indicate statistically significant differences of at least (*p* < 0.05) between treatments under the same culture conditions, while (*) indicate statistical differences between the same treatment under different culture environments during IVM (***p < 0.001) (****p < 0.0001).

Since GC-EVs enhanced the proper function and overall quality of bovine oocytes subjected to HS, we next assessed this correlation on their downstream embryonic developmental potential following *in vitro* fertilization. As shown in Figure 6A, there are no measurable differences in cleavage rates of the oocytes under thermal stress conditions, although, minimal improvements were noted for oocytes supplemented with N-EVs and cultured at 38.5°C. However, HS induces a dramatic decline in the proportion of oocytes capable of developing to the blastocyst stage, whether expressed as the percentage of oocytes developed to blastocyst (Figure 6B; *p* < 0.0001) or the percentage of cleaved embryos (Figure 6C; *p* < 0.0001). As shown in Figures 6B, C, while supplementation of S-EVs during oocyte maturation rescued the majority of oocytes developed to the blastocyst stage under thermal stress conditions, both N-EVs, and S-EVs improve the developmental rate of oocytes becoming blastocysts under normal physiological conditions, when compared to the untreated or vehicle controls. Taken together, our developmental competence data show significant differences between treatments (*p* = 0.0002, *p* = 0.0003) and temperatures (*p* < 0.0001).

### 3.5 GC-EV supplementation modifies the expression of stress-associated transcripts in blastocysts

**FIGURE 7:**
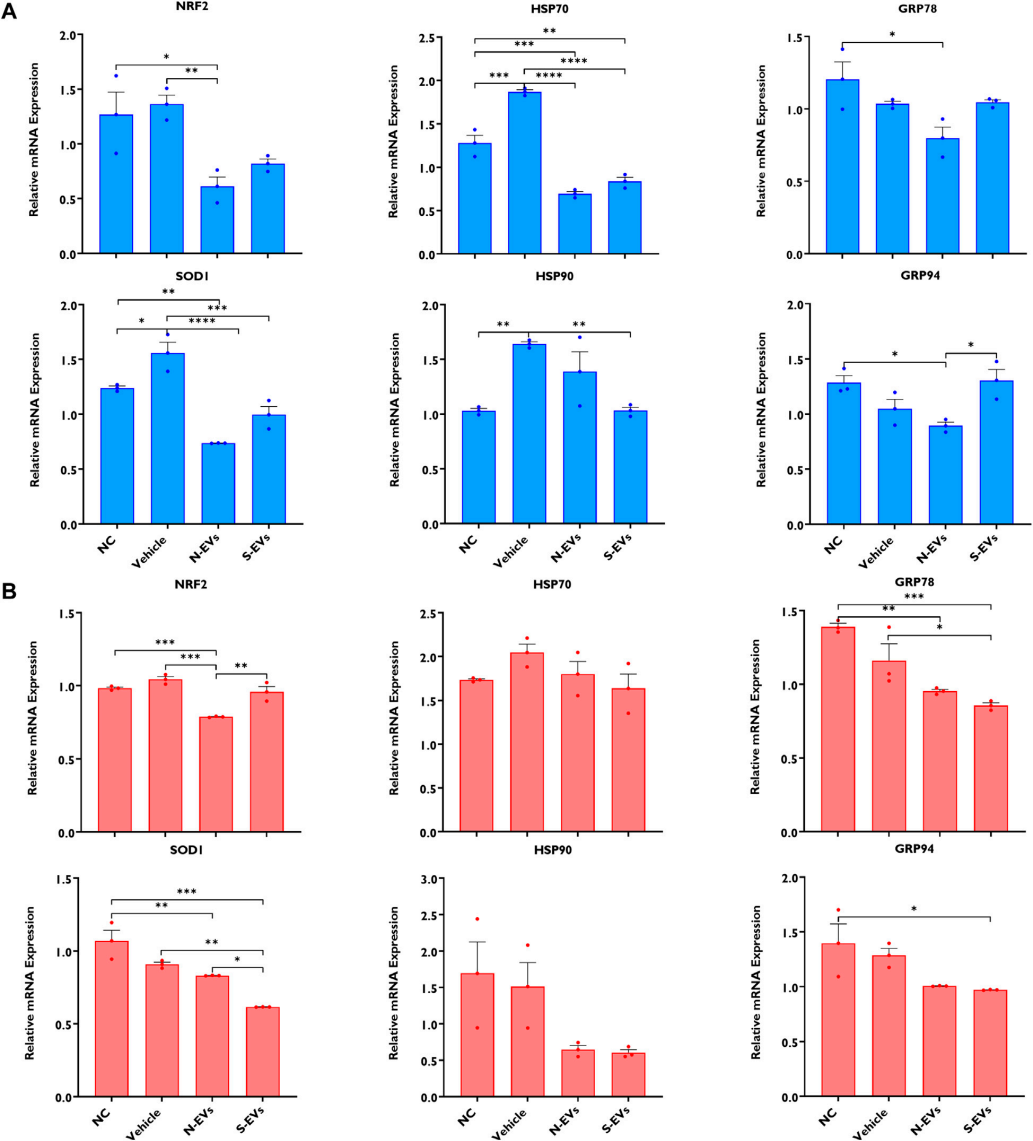
The impact of GC-EVs on transcript levels of stress-associated genes in blastocysts derived from oocytes treated with GC-EVs during oocyte maturation at 38.5°C (blue) and 41°C (red). The expressions of stress-associated genes were significantly diminished in blastocysts derived from oocytes supplemented with N-EVs and cultured at 38.5°C **(A)**, and similar decreases in gene expression were visualized in blastocysts derived from oocytes supplemented with S-EVs and exposed to HS **(B)**. Results are from a total of four pools of 5 blastocysts per pool for each treatment. Data represent the mean ± SEM and the differences between means were analyzed using One-way ANOVA followed by Tukey’s Multiple Comparisons Test. (*p < 0.05), (**p < 0.01), (***p < 0.001), (****p < 0.0001).

As shown in Figure 7A, except for GRP78 and GRP94, the supplementation of both N-EVs and S-EVs during oocyte maturation in the absence of subsequent thermal stress resulted in the cohesive suppression of the tested stress-related genes in blastocysts. Similarly, with an exception for NRF2, the supplementation of both N-EVs and S-EVs during oocyte maturation in the presence of thermal stress exposure resulted in the suppression of all six of the tested stress-associated marker genes within the resulting blastocysts (Figure 7B).

### 3.6 Impact of GC-EVs supplementation during oocyte maturation on blastocyst ROS accumulation, mitochondrial function, total cell counts, and apoptotic index

**FIGURE 8:**
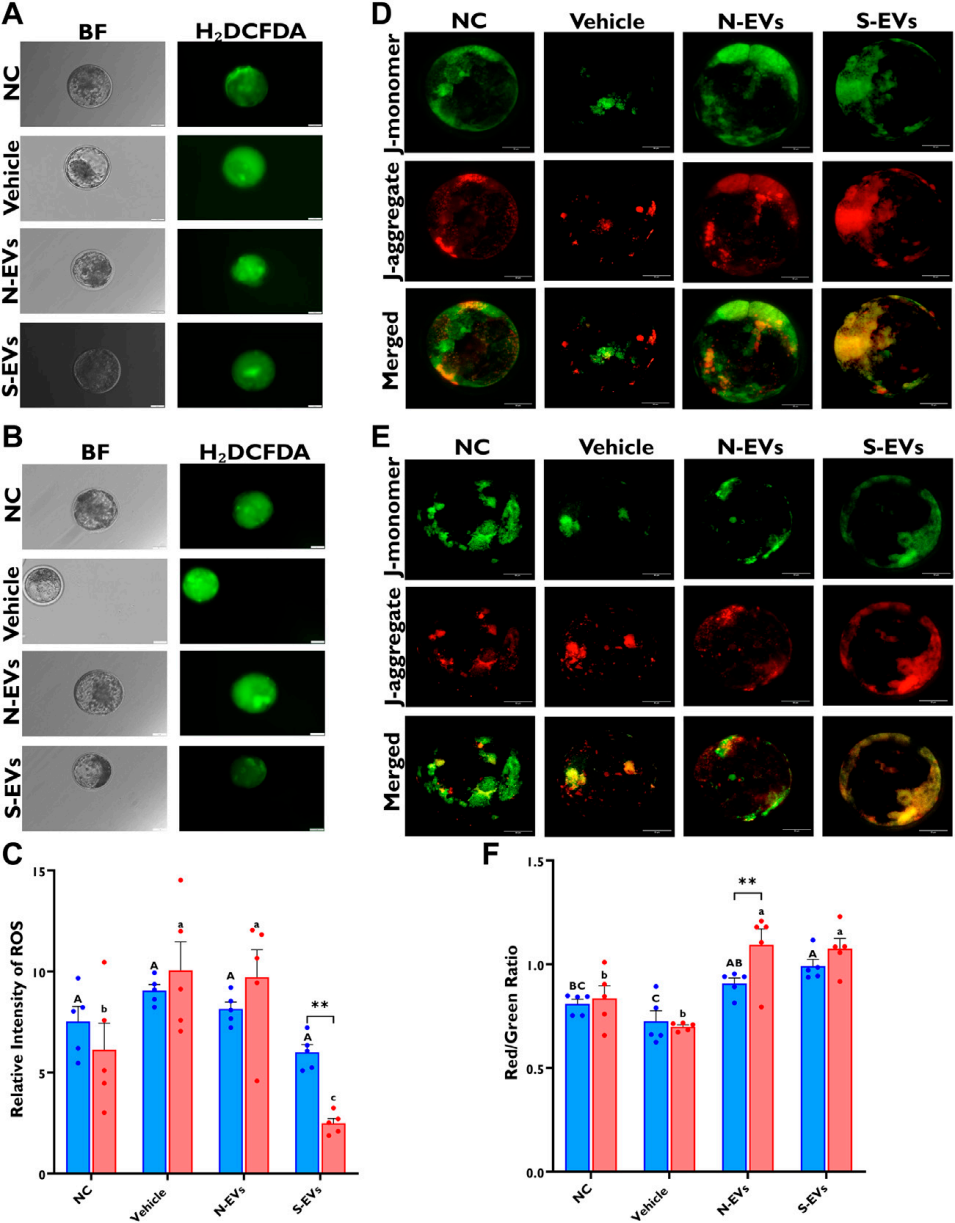
Functional alterations of GC-EV supplementation during *in vitro* maturation on blastocyst quality under *in vitro* normal physiological (38.5°C; blue) and HS (41°C; red) conditions. Fluorescence images of *in vitro* cultured bovine blastocysts treated with 2ʹ,7ʹ-Dichlorofluorescin Diacetate (H₂DCFDA) were captured for the measurement of intracellular ROS levels in single blastocysts. Scale bar, 50 µM [38.5°C; **(A)**] [41°C; **(B)**]. Quantification of the relative fluorescence intensity in single blastocysts were carried out in triplicates (a total of 6-9 blastocysts per treatment) **(C)**. Fluorescence images of *in vitro* cultured bovine blastocysts treated with 5,5′,6,6′-tetrachloro-1,1′,3,3′tetraethylbenzimidazolylcarbocynanine iodide (JC-1) for the measurement of MMP (∆Ψm), an indicator of mitochondrial activity in single blastocysts. Scale bar, 50 µM [38.5°C; **(D)**] [41°C; **(E)**]. Quantification of the relative fluorescence intensity (red/green ratio) in single blastocysts were carried out in duplicates (a total of 7–11 blastocysts per treatment) **(F)**. Data among treatments represent the mean ± SEM and the differences between means were analyzed using Two-way ANOVA followed by Tukey’s Multiple Comparisons Test. Bars with different letters (38.5°C; uppercase) (41°C; lowercase) indicate statistically significant differences of at least (*p* < 0.05) between treatments under the same culture conditions, while (*) indicate statistical differences between the same treatment under different culture environments during IVM (**p < 0.01).

As shown in Figures 8B, C, both qualitative and quantitative ROS levels are markedly lower in blastocysts when S-EVs are supplemented during oocyte maturation under HS exposure. Similarly, when cultured at 38.5°C, blastocysts derived from oocytes supplemented with S-EVs had a significantly lower accumulation of ROS (Figures 8A, C). Although our data show no significant differences between temperatures, we show a significant difference among treatments (*p* = 0.001) with a significant interaction between temperature and treatment (*p* = 0.0023). We also assessed the membrane potential of mitochondria amid blastocysts and revealed that under HS conditions, blastocysts derived from both N-EVs and S-EVs supplemented groups had significantly increased mitochondrial function, as evidenced by an increased ratio of fluorescence imaged from J-aggregates vs. J-monomers compared to the NC and vehicle (Figures 8E, F). In parallel, blastocysts derived from oocytes supplemented with N-EVs and S-EVs and cultured under normal physiological temperature (38.5°C) showed improved mitochondrial membrane potential compared to the NC and vehicle supplemented groups (Figure 8D). Collectively, our data demonstrate a significant difference between GC-EV supplemented treatments (*p* = 0.0001), culture temperatures (*p* = 0.0054), as well as their interaction (*p* = 0.0161).

**FIGURE 9:**
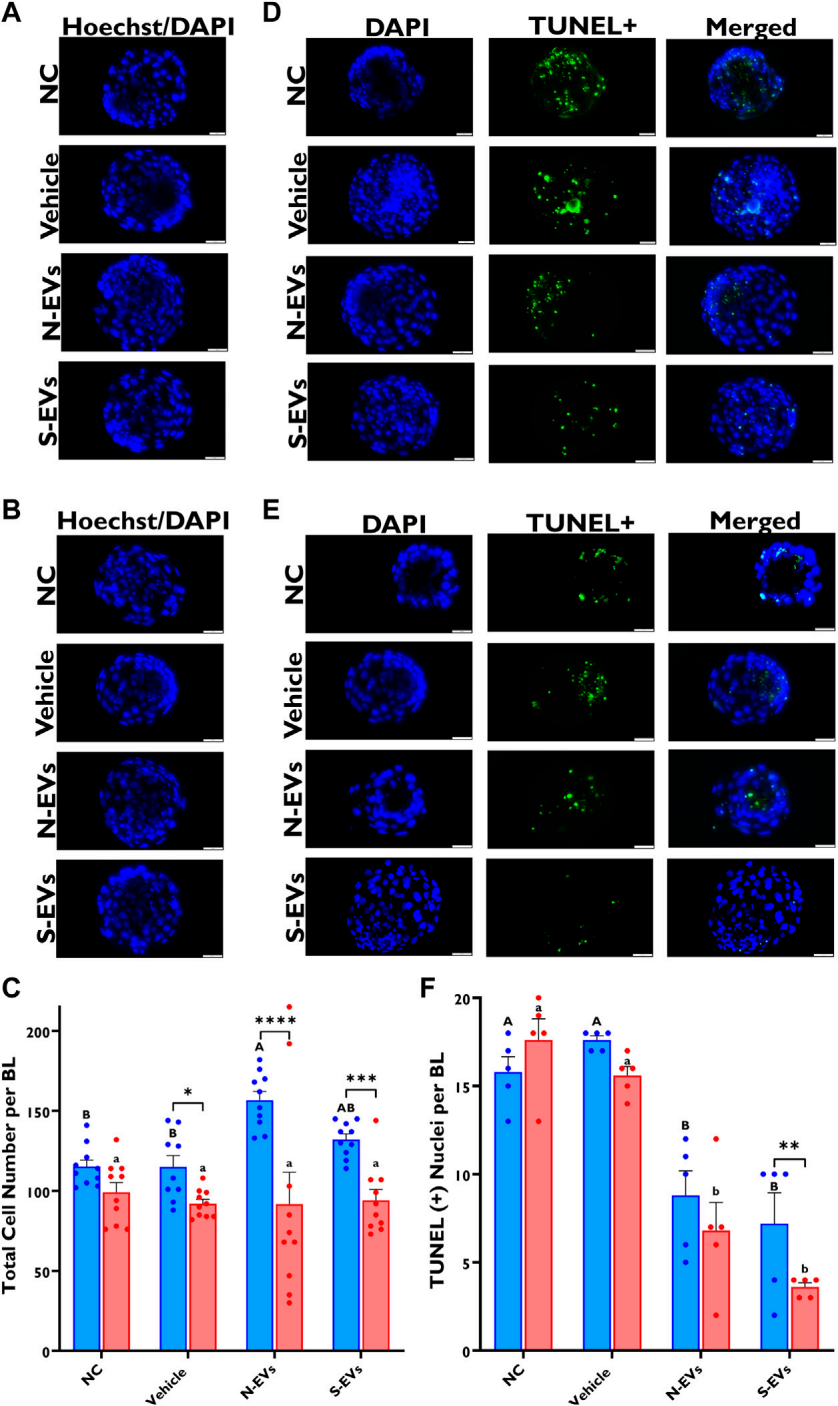
The beneficial effect of GC-EV supplementation during *in vitro* maturation on blastocyst quality under *in vitro* normal physiological (38.5°C; blue) and HS (41°C; red) conditions. Fluorescence images of *in vitro* cultured bovine blastocysts treated with Hoechst 33342/DAPI were used to determine total cell numbers in preimplantation embryos. Scale bar, 50 µM [38.5°C; **(A)**] [41°C; **(B)**]. Quantification of total cell numbers in single blastocysts were carried out in triplicates (a total of 18–30 blastocysts per treatment) **(C)**. Fluorescence images of *in vitro* cultured bovine blastocysts subjected to a TUNEL assay were captured for specific detection of apoptotic cells. Scale bar, 50 µM [38.5°C; **(D)**] [41°C; **(E)**]. Quantification of TUNEL + cells in single blastocysts were carried out in triplicates (a total of 19–41 blastocysts per treatment) **(F)**. Data among treatments represent the mean ± SEM and the differences between means were analyzed using Two-way ANOVA followed by Tukey’s Multiple Comparisons Test. Bars with different letters (38.5°C; uppercase) (41°C; lowercase) indicate statistically significant differences of at least (*p* < 0.05) between treatments under the same culture conditions, while (*) indicate statistical differences between the same treatment under different culture environments during IVM (*p < 0.05), (**p < 0.01), (***p < 0.001), (****p < 0.0001).

Alternatively, no measurable differences were observed in total cell numbers among the treatment groups subjected to HS (Figures 9B, C). However, there were significant increases in the total cell number in blastocysts derived from oocytes supplemented with N-EVs and cultured at 38.5°C (Figures 9A, C). In terms of total cell counts, we show no significant differences between treatments, albeit significant differences between temperatures (*p* < 0.0001), as well as a significant interaction between temperature and treatment (*p* = 0.0015). Moreover, as shown in Figures 9E, F, the number of fluorescently labeled nuclei with fragmented DNA (shown in green), is dramatically reduced (*p* < 0.0001) in blastocysts derived from oocytes supplemented with S-EVs and exposed to HS. Additionally, blastocysts derived from oocytes supplemented with N-EVs and S-EVs and cultured under normal physiological temperature (38.5°C) showed diminished apoptotic indexes (Figures 9D, F). Importantly, our data indicates a significant difference among treatments (*p* < 0.0001), temperatures (*p* = 0.0057), as well as their interaction (*p* = 0.0048).

## 4 Discussion

Elevated ambient temperatures play a multifaceted role in affecting physiological and cellular functions with direct detrimental impacts on fertility in dairy cattle (Wolfenson et al., 2000). Delineated in a plethora of studies, HS compromises reproductive function by altering follicular dynamics (Badinga et al., 1993; Guzeloglu et al., 2001), afflicting proper granulosa cell function (Li et al., 2016) with impairments in oocyte maturation, fertilization (Roth and Hansen, 2005; Nabenishi et al., 2012) and preimplantation embryo development (Al-Katanani et al., 2002; Roth and Hansen, 2004). Oxidative stress, modeled through HS, is evidenced by the harmful effects elicited at the cellular level by inducing DNA damage, protein modifications, and lipid peroxidation (Alvarez, 2003; Kitagawa et al., 2004). We and others have recently shown that GCs exposed to HS release EVs containing protective signals which can be beneficial in eliciting positive responses or increased protection against stress in recipient or bystander cells (Bewicke-Copley et al., 2017; Gebremedhn et al., 2020). Hence, given the physiological relevance of the whereabouts of the oocyte *in vivo* at the time of maturation, in the present study, we generated subsets of EVs released from *in vitro* cultured follicular GCs subjected to the normal ambient body temperature of the cow (38.5°C) and HS (42°C) conditions. Throughout this work, we sufficiently demonstrate the capacity of GC-derived N-EVs and S-EVs in modulating a multitude of factors associated with oocyte quality when cultured under suboptimal conditions.

It has been shown previously that the addition of follicular fluid exosomes to the maturation medium can increase cumulus expansion with projections to modulate developmental competence under HS conditions (Rodrigues et al., 2019). In addition, exosomes isolated from bovine follicular fluid of small follicles have been reported to support cumulus expansion of bovine and mouse COCs cultured without fetal calf serum (Hung et al., 2015). Similarly, we found that GC-derived EVs can support cumulus expansion in COCs during maturation in the presence or absence of thermal stress. This was evidenced by the induction of the expression of cumulus expansion-associated genes (PTGS2, EGFR, and PTX3) in COCs supplemented with GC-EVs and subsequently subjected to thermal stress compared to the NC and vehicle groups. Interestingly, the impact of N-EVs in inducing the expression of cumulus expansion marker genes was prominent in COCs cultured under normal conditions following EV supplementation (Figure 2B). On the contrary, S-EVs suppressed the expression of cumulus expansion marker genes in the absence of HS (Figure 2C). Statistically comparing treatments under differing ambient culture environments, we collectively show the beneficial effects of N-EVs under normal physiological temperature compared to HS, while S-EVs showed similar effects on cumulus expansion under HS *versus* normal temperature. Moreover, expression analysis of stress-associated genes in CCs and oocytes revealed the role of GC-derived EVs in reducing the expression of stress-associated transcripts, thereby the severity of thermal stress in those cells. Irrespective of environmental culture conditions, S-EVs in particular, clearly repressed the expression of HSP70, HSP90, NRF2, and SOD1 in CCs, signifying the inherent presence of other stress factors in our *in vitro* culture environment, even under normal culture conditions. To date, the precise molecular pathways in which GC-EVs promote thermotolerance in COCs are not fully understood. However, it is postulated that donor cell cytoplasmic proteins and RNA-enriched EVs can have the potential to modulate the stress response mechanisms of recipient cells. Moreover, EVs are widely accepted for their physiochemical traits to foster therapeutic applications primarily on EV-delivered bioactive compounds (mRNA, miRNA, long non-coding RNAs, DNAs, proteins, and lipids) with biological regulatory functions (Liu et al., 2022). Exosome-mediated transfer of miRNA in bovine follicular GCs has been evidenced in our previous study (Sohel et al., 2013). Collectively, as it has been demonstrated in previous studies (Mothersill and Seymour, 1997; Lyng et al., 2000; Chai and Hei, 2008; Bewicke-Copley et al., 2017), results from this study demonstrate the capabilities of GC-EVs to support cumulus expansion and conventional physiology underpinning proper oocyte function by reducing the severity of thermal stress during *in vitro* oocyte maturation.

Oocyte competence is largely determined by the quality and subsequent accumulation of organelles, metabolites, and maternal RNAs acquired during the period of growth and maturation determining the success of fertilization, embryo development, and pregnancy establishment. Following confirmation of GC-EVs’ impact on CCs, we examined the expression of stress-associated transcripts in the presence or absence of subsequent thermal stress. Under HS exposure, the expression of NRF2, SOD1, HSP70, HSP90, GRP78, and GRP94 was dramatically reduced in oocytes supplemented with S-EVs. These observations are similar to our previous report showing that priming of naïve GCs using EVs derived from thermally stressed bovine GCs could provide protection against thermal stress by suppressing the expression of stress-associated genes as a means of inducing thermotolerance (Gebremedhn et al., 2020). Previous reports well document overexpressed levels of HSPs to be especially detrimental to cells undergoing developmental growth and rapid divisions (Krebs and Feder, 1997). The chaperoning functions of HSPs inhibit Caspase processing, a heat-sensitive target leading to heat shock-induced apoptosis (Mosser et al., 1997). Inhibition of heat-shock-induced apoptosis using a caspase inhibitor has been shown to effectively mitigate the detrimental impacts of HS on oocyte competence for fertilization and subsequent embryo development (Roth and Hansen, 2004). Taken together, as functionally evidenced in part by others, S-EVs inherently contain high expression of HSPs (Gebremedhn et al., 2020), which likely in part inhibit caspase activity and provide resistance to stress-induced apoptosis, modulating the thermoprotective role of the CCs (Tatemoto et al., 2000) surrounding the oocyte and perhaps the integrity and function of the oocyte under HS conditions. In addition, S-EVs’ inherent capacity to induce a beneficial “bystander effect,” may potentially underpin the transfer of specific cargoes (possibly miRNA transfer), which serve to post-transcriptionally regulate gene expression *via* degradation and/or translational repression. Conclusively, S-EV-coupled miRNAs may be involved in the posttranscriptional regulation of HSPs under thermal stress conditions, resulting in the downregulation of HSP transcripts compared to the control counterparts.

Exposure to thermal stress is known to have direct implications on cellular function as it leads to the accumulation of intracellular ROS causing oxidative damage (Azad et al., 2010), mitochondrial dysfunction, and autophagy (Scherz-Shouval and Elazar, 2011; Shen et al., 2017), resulting in a continued decline in fertility (Guérin et al., 2001). The overwhelming importance of mitochondria for the development of oocytes and embryos as an apoptotic regulator and importantly a prominent source of energy in the form of ATP is widely accepted (van Blerkom, 2009). Mitochondrial dysfunction has been suggested to be a major factor in chromosomal anomalies during meiotic divisions in oocytes and mitosis in embryos affecting the developmental competence of oocytes and embryos (Schon et al., 2000; Eichenlaub-Ritter et al., 2004; Desler et al., 2007). Studies in mice and humans using JC-1 have shown the relationship between mitochondrial activity and the developmental competence of oocytes and embryos (Acton et al., 2004). In addition to reductions in ROS accumulation, supplementation of S-EVs at the time of oocyte maturation and subsequent exposure to HS, notably increased the ratio of red to green fluorescence after JC-1 staining, indicating improved mitochondrial activity. On the other hand, under normal physiological temperatures, GC-EV supplementation synergistically reduced the accumulation of ROS with the highest mitochondrial activity coming from oocytes derived from the treatment supplemented with N-EVs during IVM. Comparisons between treatments under differing ambient culture environments showed increased mitochondrial activity in oocytes supplemented with N-EVs under normal physiological temperature compared to HS conditions. Similarly, the supplementation of S-EVs also had a similar impact on mitochondrial function in oocytes under HS conditions *versus* normal ambient temperature. The positive impacts of GC-EVs during oocyte maturation in the absence of thermal stress exposure may be in part due to the intrinsic quality of oocytes derived from local slaughterhouse ovaries and indicative that certain levels of stress are inherently present within the *in vitro* environment.

To investigate the downstream effect of GC-EVs supplementation during bovine IVM, we performed IVF and IVC of the resulting oocytes for development until the blastocyst stage. Supplementation of both N-EVs and S-EVs during oocyte maturation showed to improve blastocyst rates both in the presence or absence of thermal stress exposure, indicating the positive impact of GC-EVs in supporting oocyte development to the blastocyst stage (Figures 6B, C). Moreover, a significant reduction in the expression of stress-associated genes (SOD1, HSP70, HSP90, GRP78, and GRP94) was observed in blastocysts derived from COCs supplemented with N-EVs and S-EVs. However, in the absence of HS, blastocysts from GC-EVs’-supplemented treatment groups tended to suppress the relative abundance of stress-associated genes, with exceptions to the expression of GRP78 and GRP94, which showed increases in expression in S-EVs supplemented treatment groups. This indicates the differential impact of GC-EVs in modulating the stress response of oocytes to environmental stressors. In the present study, it is shown that blastocysts derived from COCs supplemented with S-EVs and exposed to HS during maturation have increased embryo quality due to reduced ROS accumulation, decreased TUNEL+ apoptotic nuclei, and increased mitochondrial activity compared to their NC counterparts. Interestingly, as shown in oocytes, S-EVs significantly reduced the amount of accumulated ROS in blastocysts under HS compared to those cultured under normal ambient temperature. These results partly support our hypothesis that S-EVs derived from GCs mitigate the detrimental effects of HS during oocyte maturation, which may have an impact on the further developmental potential of oocytes in giving rise to developmentally competent blastocysts. Our data also suggests that GC-EV supplementation amid HS exposure during IVM may not induce the proliferative capacity of bovine embryos, however, GC-EVs effectively modulate the inherent quality of resulting blastocysts. To leverage our findings, a similar study showed that EVs isolated *via* density gradient ultracentrifugation and size-exclusion chromatography from Holstein heifer follicular fluid, improve embryo quality based primarily on differential apoptotic cell counts (Asaadi et al., 2021). Similarly, the addition of bovine oviduct epithelial cell EVs had a positive impact on the quality of *in vitro* produced bovine embryos, signifying EVs’ functional and physiological relevance to support the development of viable and competent blastocysts under *in vitro* environments to enhance the pregnancy outcome after transfer (Lopera-Vásquez et al., 2016).

**FIGURE 10:**
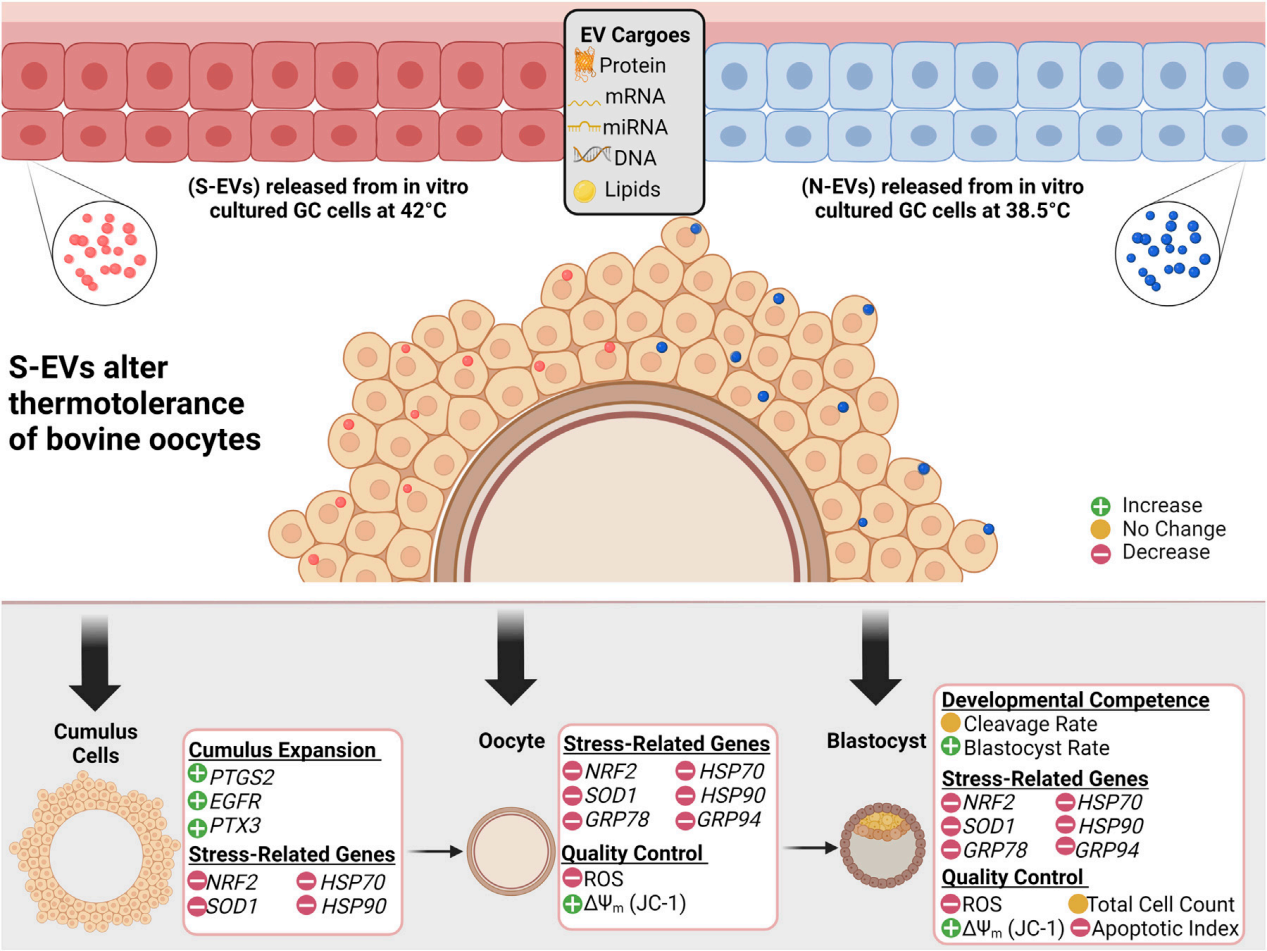
Graphical Synopsis. Schematic illustration showing EVs secreted from follicular granulosa cultured *in vitro* under 38.5°C (N-EVs) and 42°C (S-EVs), preferentially uptaken in the cumulus cells in intact cumulus-oocyte complexes following co-incubation during *in vitro* oocyte maturation. Results indicate that S-EVs effectively modulate the thermotolerance of bovine oocytes to elevated temperatures shown through the functional analysis conducted in the cumulus cells, oocytes, and developed blastocysts.

Taken together, the current study demonstrated that GC-EVs play a pivotal regulatory role against HS during *in vitro* maturation of bovine oocytes by reducing ROS accumulation, enhancing proper mitochondrial function, and altering the expression of stress-associated genes, thereby reducing the severity of thermal stress, improving oocyte survival and viability (Figure 10). Despite the fact that EVs are uptaken only by CCs, the impact of EV supplementation seems to be through changing the physiology of the CCs, which ultimately lends impact on oocyte physiology, particularly ROS accumulation, mitochondrial activity, and gene expression profiles. Furthermore, the beneficial role of EVs and their capabilities to modulate the detrimental effects of HS during maturation can have long-lasting impacts on the development and quality of blastocysts, potentially in the establishment of pregnancy and the generation of healthy offspring. As GC-EVs are known to shuttle protective signals in the form of RNAs, proteins, and/or lipids, further studies are needed to determine the exact mechanisms of action of EVs in modulating stress response for potential application in assisted reproductive technologies involving IVM, IVF, and IVC of mammalian oocytes and embryos.

## Data Availability

The original contributions presented in the study are included in the article/Supplementary Material, further inquiries can be directed to the corresponding author. Written details on experimental procedures have been submitted to the EV-TRACK knowledgebase and can be accessed under (EV-TRACK ID: EV220412).
