# Supplementary material for: Granulosa cell-derived extracellular vesicles mitigate the detrimental impact of thermal stress on bovine oocytes and embryos
Source: Front Cell Dev Biol. 2023 Apr 6;11:1142629. doi: 10.3389/fcell.2023.1142629 (PMC10116072; doi:10.3389/fcell.2023.1142629)
Supplement: Supplementary file 1 [file Table1.docx]

| **Target** | **Catalog #** | **Dilution** | **Manufacturer** |
| --- | --- | --- | --- |
| Anti-CD63 | EXOAB-CD63A-1 | 1:100 | System Biosciences |
| Anti-TSG101 | EXOAB-TSG101-1 | 1:100 | System Biosciences |
| Anti-CD81 | EXAB-CD81A-1 | 1:100 | System Biosciences |
| Cytochrome c | 102139-T42 | 1:200 | Sino Biological |

**Supplementary Table S1**. List of Antibodies used.
