# Supplementary figures and images for: Granulosa cell-derived extracellular vesicles mitigate the detrimental impact of thermal stress on bovine oocytes and embryos
Source: Front Cell Dev Biol. 2023 Apr 6;11:1142629. doi: 10.3389/fcell.2023.1142629 (PMC10116072; doi:10.3389/fcell.2023.1142629)

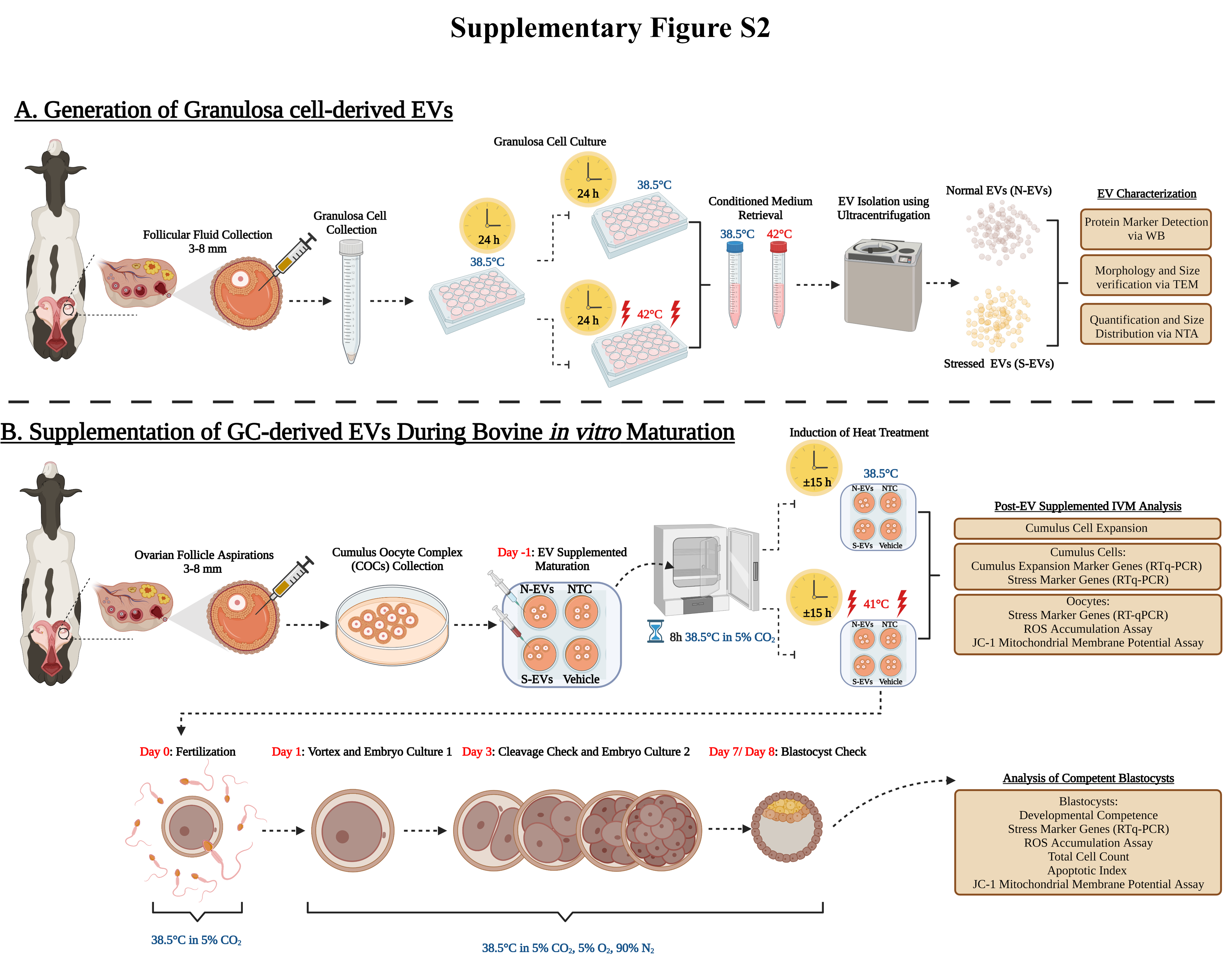

Supplement: Supplementary file 2 [file Image2.tif]

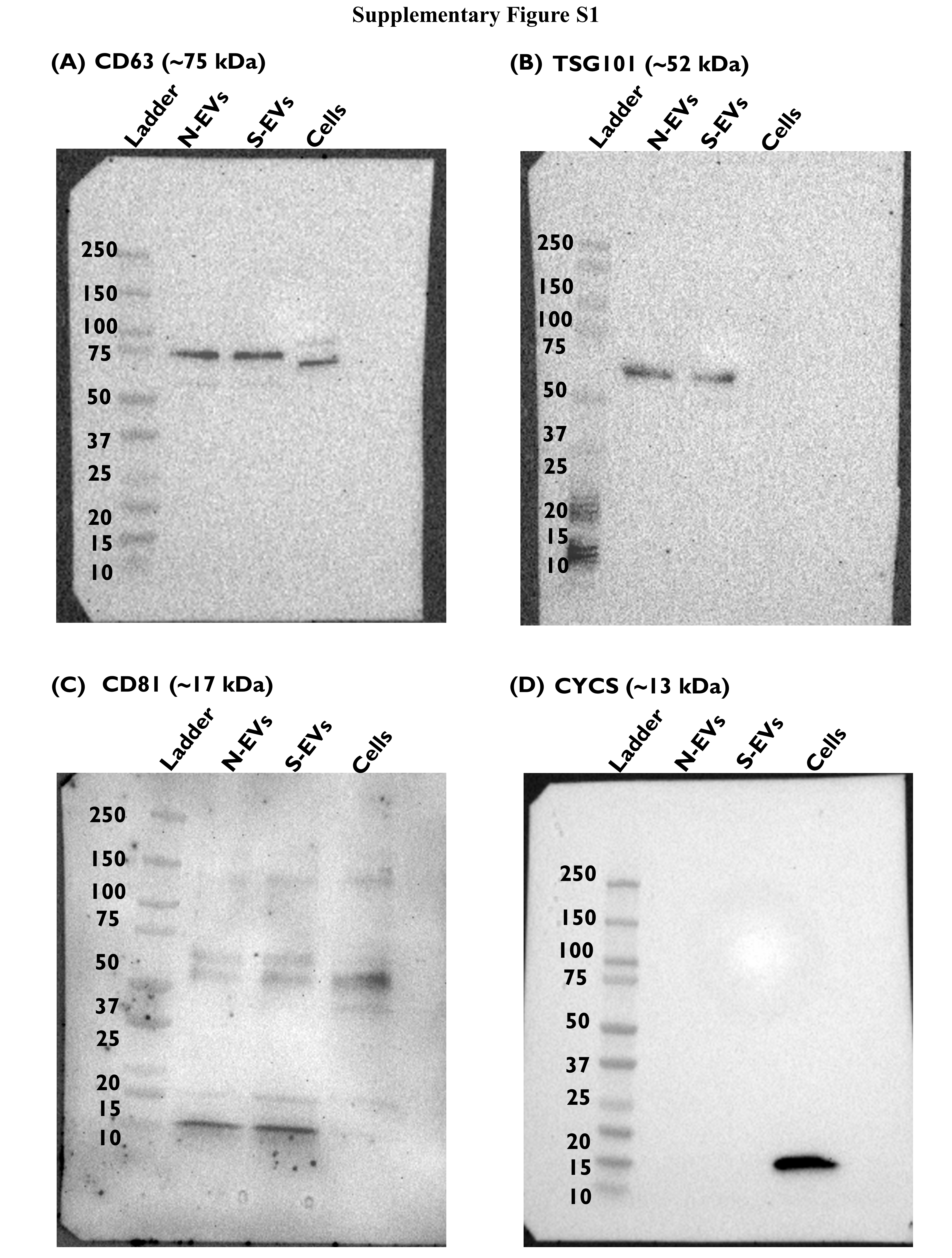

Supplement: Supplementary file 3 [file Image1.tif]
