# Supplementary material for: Granulosa cell-derived extracellular vesicles mitigate the detrimental impact of thermal stress on bovine oocytes and embryos
Source: Front Cell Dev Biol. 2023 Apr 6;11:1142629. doi: 10.3389/fcell.2023.1142629 (PMC10116072; doi:10.3389/fcell.2023.1142629)
Supplement: Supplementary file 4 [file Table2.docx]

| **Gene name** | **Accession number** | **Primer sequences** |
| --- | --- | --- |
| PTGS2 | NM_174445 | F5`-cgatgagcagttgttccaga-3´  R5´-gaaagacgtcaggcagaagg-3´ |
| EGFR | XM_002696890 | F5`-gacccgaaagaactggacat-3´  R5´-tgttatatccaggccgacaa-3´ |
| PTX3 | NM_001076259 | F5`-acctgggattcaaagaaagg-3´  R5´-caccctcccagatattgaag-3´ |
| NRF2 | NM_001011678 | F5´-cccagtcttcactgctcctc-3´  R5´-tcagccagcttgtcattttg-3´ |
| SOD1 | NM_174615 | F5´-agaggcatgttggagacctg-3´  R5´-cagcgttgccagtctttgta-3´ |
| HSP70 | NM_001038505 | F5´-aatgccagttgccaatgctg-3´  R5´-atcgagagttcctccaccca-3´ |
| HSP90 | NM_001012670 | F5´-tcactgaggaaatgccaccc-3´  R5´-atggagacagagcgctgaac-3´ |
| GRP78 | NM_001075148 | F5´-tgcgaagccctatagctgac-3´  R5´-agtaggtggtacccaggtcg-3´ |
| GRP94 | NM_174700 | F5´-tgctgtgtggagagggaatg-3´  R5´-tcctgtgaccacaatcccaa-3´ |
| β-ACTIN | NM_173979 | F5´-tgtccaccttccagcagat-3´  R5´-tcaccttcaccgttccagt-3´ |
| GAPDH | NM_001034034 | F5´-aatggagccatcaccatc-3´  R5´-gtggttcacgcccatcaca-3´ |

**Supplementary Table S2**. Sequence specific primers used for qRT-PCR analysis.
